# Quick conversions and de novo synthesis within the entire α- and β-carotenoid branches during non-steady-state light transients

**DOI:** 10.1007/s11120-026-01221-5

**Published:** 2026-06-06

**Authors:** Sara Pescador-Dionisio, Adrián Moncholí-Estornell, Mª Pilar Cendrero-Mateo, Eva Neuwirthová, María Jesús Rodrigo, Lorenzo Zacarías, José Moreno, Shari Van Wittenberghe

**Affiliations:** 1 Laboratory for Earth Observation, Image Processing Laboratory, University of Valencia, C/Catedràtic Agustín Escardino Benlloch, Paterna, Valencia 46980 Spain; 2 Institute of Agrochemistry and Food Technology, IATA-CSIC, C/Catedràtic Agustín Escardino Benlloch, 7, Paterna, Valencia 46980 Spain

**Keywords:** Antenna stoichiometry, Xanthophyll cycle, Non-photochemical quenching (NPQ), Rapid light transition, Chlorophyll fluorescence, α- and β-branch carotenoids

## Abstract

Understanding the rapid adjustments of plants to high-light exposure remains challenging, as multiple excitation and de-excitation pathways are simultaneously activated. In this study, we examined carotenoid pigment conversions at the second-scale in three tree species in parallel with high temporal resolution (<1 s) in vivo fluorescence and absorption spectroscopy. Our results reveal that both β-branch (violaxanthin, antheraxanthin, zeaxanthin) and α-branch (lutein, lutein epoxide) xanthophylls exhibit remarkably fast and oscillating pool dynamics within the first 20 seconds of illumination, reaching even maximal values in that timeframe. Prompt (0–20 s) conversion of the lutein is observed at the expense of both lutein epoxide and α-carotene in certain species, while accumulation of antheraxanthin and zeaxanthin is seen both prompt (0–20 s) and slower (>30 s). Interestingly, mirror trends between whole α- and β-branch carotenoids seem to indicate balancing trends, involving dynamic precursor shifts between α- and β-carotenes. Further, we observe that quick xanthophyll changes match the kinetic trends of fitted Gaussian-modeled absorbance peaks (approx. at 520, 535, 560 nm) within the early seconds. These quick changes in photon absorption are followed by slower-triggered non-photochemical de-excitation through a particular xanthophyll, seen from the dominant 535-nm peak, and likely attributed to antheraxanthin or zeaxanthin. The quick xanthophylls conversion redistributing the excessive excitation energy while quenching fluorescence (EET phase) is shown as one of the first responses to excessive light, before regulated energy dissipation as heat is initiated. These observations invite to interpret the non-steady state conditions and their parametrization more carefully, considering different photoprotective strategies across species.

**Supplementary Information:**

The online version contains supplementary material available at 10.1007/s11120-026-01221-5.

## Introduction

Millions of years of evolution have produced extraordinary adaptations and solutions for plants to face naturally excessive solar energy which commonly cannot be fully utilized by the light harvesting apparatus of leaves. To find a balance between the harvesting of, and the protection against the solar radiation conditions, all plants employ flexible regulated heat dissipation mechanisms. The regulation of the excessive energy dissipation is a very dynamic concept within the photosynthetic antenna through several heterogeneous processes taking place at different timescales (Holzwarth et al. 2009; Lambrev et al. 2012). It activates to maintain optimal photochemical energy quenching in accordance with environmental conditions. A simple model proposed by Butler (1978) was the origin of developing equations used for fluorimeters to detect fluorescence (F) yield changes enabling the nondestructive, quantitative determination of photochemical versus nonphotochemical processes in leaves. This original model predicted that photosystem II (PSII) fluorescence emission could be used to monitor changes in photochemistry, provided that the rate constants for fluorescence and regulated heat loss do not change (Baker 2008). Later, new fluorescence parameters were developed considering the variable non-photochemical energy dissipation, while still keeping assumed constant rate constants for fluorescence (Kramer et al. 1995, 2004b, c; Krause et al. 1980; Krause and Jahns 2004). Hence, fluorescence changes have been commonly interpreted as changes in both photochemical quenching and in non-photochemical quenching (NPQ). The latter is assumed to be only assessed through the application of saturating pulses, which close the reaction centers (RC) and cancel out any fluorescence change due to photochemical quenching (Laisk et al. 1997). When a dark-adapted leaf is suddenly exposed to strong illumination, an initial rapid fluorescence increase is followed by a slower decline. This slow decline has been interpreted as an increasing number of processes, both on the photochemical as on the non-photochemical side (Schreiber 2004). However, even until now fundamental ambiguity among the processes and their triggers remains.

Several explanations have been given to the slow fluorescence decline, but some of them remain controversial. The fluorescence decline (requiring O_2_) has been considered to reflect the activation of photosynthetic electron transport (Kautsky and Franck 1943; Schreiber et al. 1971), and a slower re-opening of PSII reaction centers compared to photosystem I (PSI) within the first seconds of light exposure through activation of linear electron transport (leading to photochemical quenching). This interpretation is further complicated by accumulating evidence demonstrating that fluorescence carries far more information about functional activity, conformational states and structural dynamics of PSII than previously thought (Garab 2025).

In the meantime, the proton gradient across the thylakoid builds up due to electron transport-driven proton translocation into the thylakoid lumen, primarily via water oxidation at PSII and plastoquinol (PQH₂) oxidation at the cytochrome b₆f complex, which collectively contribute to the acidification of the lumen. This acidification is proposed to activate the enzyme violaxanthin de-epoxidase (VDE) and PsbS by protonation, the activity of which leads to a decrease in the efficiency of exciton delivery to the PSII centers (Klughammer et al. 2013; Kramer et al. 2003, 2004a; Li et al. 2002). It was further proposed that plants use PsbS, a pigment-free light-harvesting complex (LHC) protein of PSII, to sense the lumen pH and initiate the reversible non-photochemical energy quenching (qE) process accordingly (Li et al. 2004). Protonated PsbS in combination with available antheraxanthin (Ant) and zeaxanthin (Zea) are proposed to lead to the “turn on” of qE (Bennett et al. 2018). On the contrary, it was also demonstrated that qE can be induced in Arabidopsis *npq4* mutants lacking PsbS (Johnson and Ruban 2011; Saccon et al. 2020).

In parallel, the activation of VDE (also suggested through protonation) starts quick chemical conversions in the xanthophyll (Xan) pools, which attach to the LHCs and are required to go from a light-harvesting state to an energy quenching state (Duffy and Ruban 2015). Xanthophylls conversions are found in two branches: the β-branch, which comes from β-carotene (β-Car) and consists of violaxanthin (Vio), Ant and Zea (the VAZ cycle); and the α-branch, which comes from α-carotene (α-Car) and consists of lutein (Lut) and lutein epoxide (Lx) (the LxL cycle). Despite both xanthophyll cycles are catalyzed by the same enzymes - VDE and zeaxanthin epoxidase (ZEP)- (Demmig-Adams 1990; García-Plazaola et al. 2007), different kinetics in the VAZ and LxL cycles have been described by several authors. Their main kinetic difference is the rate of epoxidation in the dark, proposed to be much slower in the LxL cycle than in the VAZ cycle, possibly due to a lower affinity of ZEP for Lut, whereas light-induced de-epoxidation of Lx and Vio is suggested to proceed in the same way (García-Plazaola et al. 2002; Matsubara 2004; Snyder et al. 2005). On the contrary, Jia et al. (2013) observed the start of de-epoxidation of Vio and Lx in avocado leaves immediately in sunlight, but it was more rapid and greater for Vio than for Lx. Commonly, this difference in epoxidation kinetics has been interpreted as Lut being more strongly bound to chlorophyll (Chl)-binding proteins than Zea (García-Plazaola et al. 2007; Ruban et al. 1999), and the LxL cycle perhaps not being as responsive to rapid changes in light as in the VAZ cycle (Matsubara 2004). Hence, while distinct roles and kinetics between the two cycles (VAZ and LxL) have been proposed, the complementarity of both photoprotection strategies remains not fully understood.

Formed xanthophylls attach to their corresponding binding sites in the antenna where they may fulfill their photoprotective role when other triggers occur (Balevičius et al. 2017; Ilioaia et al. 2011; Jahns et al. 2001; Li et al. 2021; Liguori et al. 2015). Specific binding sites have been assigned to the xanthophylls (Liguori et al. 2015; Pan et al. 2020; Xu et al. 2015), but replacements among the xanthophylls at identical binding sites have also been demonstrated (Carbonera et al. 2022; Johnson et al. 2009; Phillip et al. 2002), suggesting that multiple strategies for energy quenching can be used.

The chemical formation of new possible quenchers, their binding to the antenna (modifying the energy distribution in the antenna), the substitution amongst each other, and the activation of non-photochemical energy dissipation upon further triggers, remains however difficult to separate based on steady-state measurements. So far, correlations between the quenching behaviour and decay time of specific features obtained from ultrafast (steady-state) absorption spectroscopy remains the main evidence to support the direct quenching behaviour of the different xanthophylls, while such measurements do not focus on the biochemical (and therefore, stoichiometric) changes in the antenna (Bennett et al. 2019).

In contrast to set-ups under steady-state conditions, non-steady-state transition phases force the antenna to prepare very quickly to safely dissipate excessive energy. Hence, the onset, triggers and versatility of the different mechanisms within such short acclimation are expected to be rather complex. The prevailing picture so far is still that carotenoid de-epoxidation starts to become significant on the timescale of several minutes to tens of minutes (Bilger et al. 1989; Jahns 1995; Kress and Jahns 2017). However, some authors have measured faster chemical conversions at the same speed as fluorescence quenching (at the second scale), in parallel with subtle absorption changes (Van Wittenberghe et al. 2019).

To appreciate better the complexity of the different chemical conversions, affecting the Xan/Chl stoichiometry and eventual energy state of the antenna, we designed an experiment looking into the individual xanthophyll conversion dynamics during quick light transitions. Concretely, we aimed at studying both α- and β-branch pigment conversion at a second-scale temporal resolution. Since the biochemical conversion activity and available pool sizes may vary upon species and growing conditions, we used sun and shade-adapted leaves of three different tree species. In addition, ultrafast in vivo absorption spectroscopy was used to identify features that relate to the chemical or possible quenching behaviour.

## Materials and methods

### Tree species and sampling strategy

**Fig. 1:**
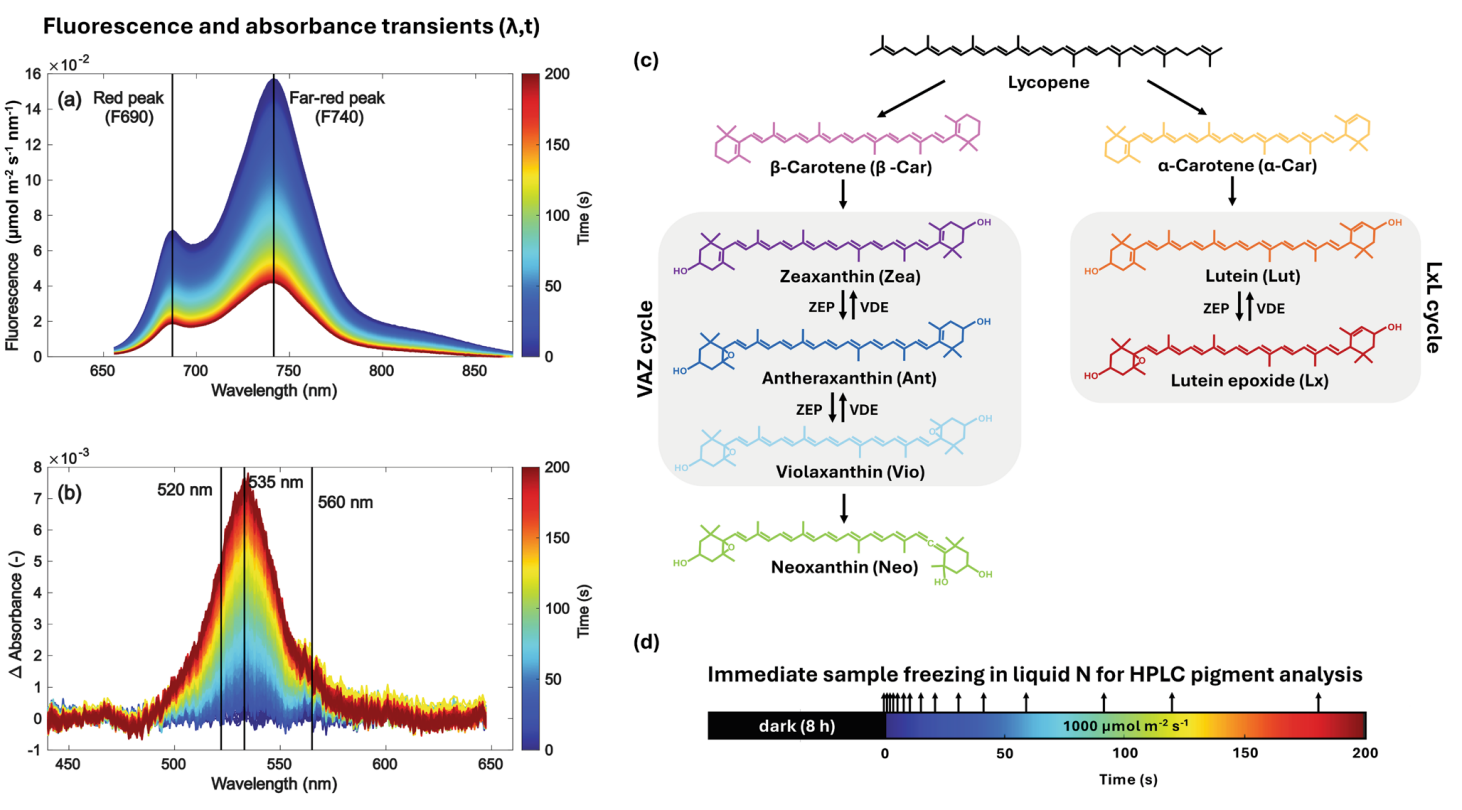
High temporal resolution leaf spectroscopy showing decreasing fluorescence (655–870 nm) and increasing absorbance (450–650 nm) changes (Δ absorbance) upon dark-to-high-light transient (λ: wavelength, t: time) (panels **a**,**b**). Wavelengths used in the analysis of fluorescence transients (F690, F740) and absorbance changes (520, 535 and 560 nm) are marked. Metabolic pathways of α- and β-carotenoid branches with the xanthophyll biosynthesis pathways illustrating the violaxanthin-antheraxanthin-zeaxanthin (VAZ) and lutein-lutein epoxide (LxL) cycles (panel **c**). Violaxanthin epoxidase enzyme (VDE) is activated in response to excessive light, switching the cycles to their photoprotective states, while zeaxanthin epoxidase (ZEP) is activated after relaxation in darkness or non-saturating light conditions. Leaf disk sampling strategy for pigment analysis at light exposure Δt along the dark-to-high-light transient is also shown (panel **d**)

We selected *Celtis australis* L. (European nettle tree, Mediterranean hackberry), *Morus alba* L. (white mulberry) and *Quercus ilex* L. (evergreen oak) as study species, with the latter known to have relatively high Lx content besides the ubiquitous VAZ pool (Fig. 1b). These species represent ecologically and phylogenetically distinct temperate woody taxa with contrasting light acclimation strategies and growth habitats, allowing assessment of whether rapid pigment dynamics are conserved across divergent perennial species rather than being restricted to herbaceous model systems.

All trees were located at the same geographic site in Paterna, Spain (39°30′58″N, 0°25′20″W). Sampling was conducted over three consecutive days (28–30 July 2023) to minimize environmental variability and branches were collected at the same time of the day to reduce potential diurnal effects. Lower shaded and upper sun-exposed branches were sampled based on canopy position and light exposure. For each species and light condition, the branches were cut and immediately submerged in water and kept in darkness at room temperature for eight hours to allow dark acclimation prior to measurements.

### Dark-to-high-light transients

Dark-to-high-light transient time series of passively emitted chlorophyll a (Chl a) fluorescence and absorbance (Fig. 1a, b) were measured in the laboratory for leaves of the three tree species collected from the field. After dark-adaptation overnight, fluorescence/reflectance/transmittance measurements were carried out using a dual VNIR (wavelength λ: 450–1100 nm) spectroradiometer (QEPro, Ocean Optics, Dunedin, FL, USA) set-up (Pescador-Dionisio et al. 2025; Van Wittenberghe et al. 2019).

From each dark-adapted branch, we sampled one or two leaves for the spectroscopy measurements during transients while they remained attached to the branch. Each leaf was clipped inside a FluoWat leaf clip (Alonso et al. 2007; Alonso Chordá 2022; Van Wittenberghe et al. 2019, 2013). The VNIR spectroradiometers, each connected to a fibre optic respectively inserted into an upward and downward leaf clip opening, were set to perform simultaneous time series measurements of the upward and downward leaf radiance (L_up_(λ, t), L_dw_(λ, t), W m^−2^ sr^−1^ nm^−1^) with a spectral resolution of ~1.5 nm in the VNIR range. Inserting a 650-nm cut-off filter at the light opening of the leaf clip further allowed the measurement in physical energy units (W m^−2^ sr^−1^ nm^−1^) of passively induced and spectrally resolved Chl a F emitted from both leaf sides, i.e. Fup(λ, t) and Fdw(λ, t), with λ=650–800 nm and *t* = 0–200 s. We used a high-voltage single LED (High Cri LED 10W 17 V 3050–5900 K, Yuji International Co., Ltd, Beijing, China), providing a 400–800 nm broadband radiation spectrum of approx. 1000 µmol m^−2^ s^−1^ in the photosynthetic active radiation (PAR) region (400–700 nm). For further details on the set-up, see Van Wittenberghe et al. (2019).

Fluorescence and absorbance transients were collected for the dark-adapted leaves exposed to sudden and constant saturating light of approx. 1000 µmol m^−2^ s^−1^ during three minutes in the spectral range of 655–850 nm (fluorescence) and 400–650 nm (absorbance), respectively. The integration time of the single acquisition was optimized for each species and in the order of 100–350 ms. After each transient protocol a white spectral reference (Spectralon, Labsphere Inc., North Sutton, USA) was placed inside the leaf clip to measure the intensity of the applied irradiance (E(λ), W m^−2^ nm^−1^) and control for the LED stability. Both the upwelling (F_up_(λ)) and downwelling (F_dw_(λ)) signals were summed to obtain the total fluorescence emission, F_tot_(λ). To assess the ^1^Chl a* F quenching kinetics, F_tot_(λ) was spectrally integrated after conversion to photon flux units to obtain the time series of the total fluorescence flux (*J*_F_) along the transient.

1 $$\begin{gathered} {J_F}\left[ {\mu mol{\mkern 1mu} {m^{ - 2}}{\mkern 1mu} {s^{ - 1}}} \right] \hfill \\ \quad \; = \int_{655}^{850} {\left( {3.14 \times {{10}^{ - 3}} \times \left( {\frac{{{{10}^6}}}{{Na}}} \right) \times \left( {\frac{{{{10}^{ - 9}} \times {\lambda _i}}}{{h \times c}}} \right) \times {F_{tot}}\left( {{\lambda _i}} \right)} \right)d\lambda } \hfill \\ \end{gathered}$$

Where $Na$ is the Avogadro’s constant ($6.02 \times {10^{23}}{\rm{mol}}{{\rm{}}^{ - 1}}$), $h$ is the Planck’s constant ($6.62{\rm{x}}{10^{ - 34}}{\rm{J}} \times {\rm{s}}$ or W $\times s \times s$), and $c$ is the speed of light (299,792,458 $m \times {s^{ - 1}}$).

These *J*_F_ values in function of time ($${t_i}$$) were used to calculate the fluorescence quenching as a proxy for the total of energy redistribution and quenching processes in the antenna. It was estimated by normalising the difference between the first fluorescence measurement at time 0 ($${t_0})$$ and the fluorescence measured at time $${t_i}$$ during the transient.

2 $$F\,quenching\left( t \right) = {{{J_F}_{\left( {{t_0}} \right)} - {J_F}_{\left( {{t_i}} \right)}} \over {{J_F}_{\left( {{t_i}} \right)}}}$$

Note that this formula is equivalent to the conventional NPQ value, calculated as (Fm-Fm’) / Fm’, with maximal fluorescence (Fm) values obtained under saturating flashes.

In addition, we normalized the overall fluorescence flux decline by calculating a normalized total fluorescence decay over time as:

3 $${\left[ {{J_F}} \right]_{norm\Delta t}}\left( t \right) = 1 - {{{J_F}_{\left( {{t_0}} \right)} - {J_F}_{\left( {{t_i}} \right)}} \over {{J_F}_{\left( {{t_0}} \right)} - {J_F}_{\left( {{t_{180}}} \right)}}}$$

To analyse further the impact of F quenching behaviour in the antenna on the fluorescence shape, we calculated the kinetic trends in the F690/F740 peak ratio. The F690/F740 ratio may indicate changes in the re-absorption due to changes in the behaviour of Chl absorption, such as conformational changes happening in the antenna, causing red-shifted emission (Miloslavina et al. 2008). Others have argued an influence of various PSI versus PSII behaviour to play a role (Sapeta et al. 2023). Other changes in the antenna affecting the F690/F740 ratio, i.e. re-absorption due to Chl loss or state-transitions (Chaux et al. 2015; Zuo 2025) were not considered significant at this short timescale.

To obtain the fluorescence peaks, we separated the fluorescence spectrum into two wavelength ranges: red (650–700 nm) and far-red (700–800 nm). We then calculated the maximum value for each range at time ${t_i}$ to obtain the fluorescence emission peaks for both.

4 $${\rm{F}}690/{\rm{F}}740{\rm{}}\left( {{t_i}} \right) = {{max\left( {{F_{tot\left( {650 - 700} \right)}}} \right)} \over {max\left( {{F_{tot\left( {700 - 800} \right)}}} \right)}}\left( {{t_i}} \right)$$

Leaf absorbance transients were computed to monitor changes in the leaf’s optical properties due to changes in the pigment pool and additional quenching regulations. The absorbance was calculated based on the assumption of energy conservation, as:

5 $${\rm{Absorbance}}\left( {\rm{\lambda }} \right) = {\rm{}}1{\rm{}} - {\rm{Transmittance}}\left( {\rm{\lambda }} \right) - {\rm{Reflectance}}\left( {\rm{\lambda }} \right)$$

Where the absorbance was tracked along the three key wavelengths 520, 535 and 560 nm. Features at these wavelengths have been spectrally monitored in previous in vivo transient experiments (Van Wittenberghe et al. 2019; García-Martínez et al. 2025) and steady-state transient absorption (TA) spectroscopy experiments. Following Van Wittenberghe et al. (2021), a Gaussian peak fitting model was applied to several absorbance transients (${\rm{\lambda }},{\rm{t}}$) to illustrate subtle absorbance changes within the first seconds upon light exposure. A spectral fitting model was applied for the λ: 450–650 nm range, using three Gaussians with respective mean wavelength (m) and standard deviation (s): G1 (*m* = 520 nm, s = 8.5 nm), G2 (*m* = 535 nm, s = 12.5 nm), G3 (*m* = 560 nm, s = 5 nm). In the fitting model, the mean was allowed to vary with ±2 nm, and the amplitude was kept free, allowing both positive and negative amplitude weights.

**Fig. 2:**
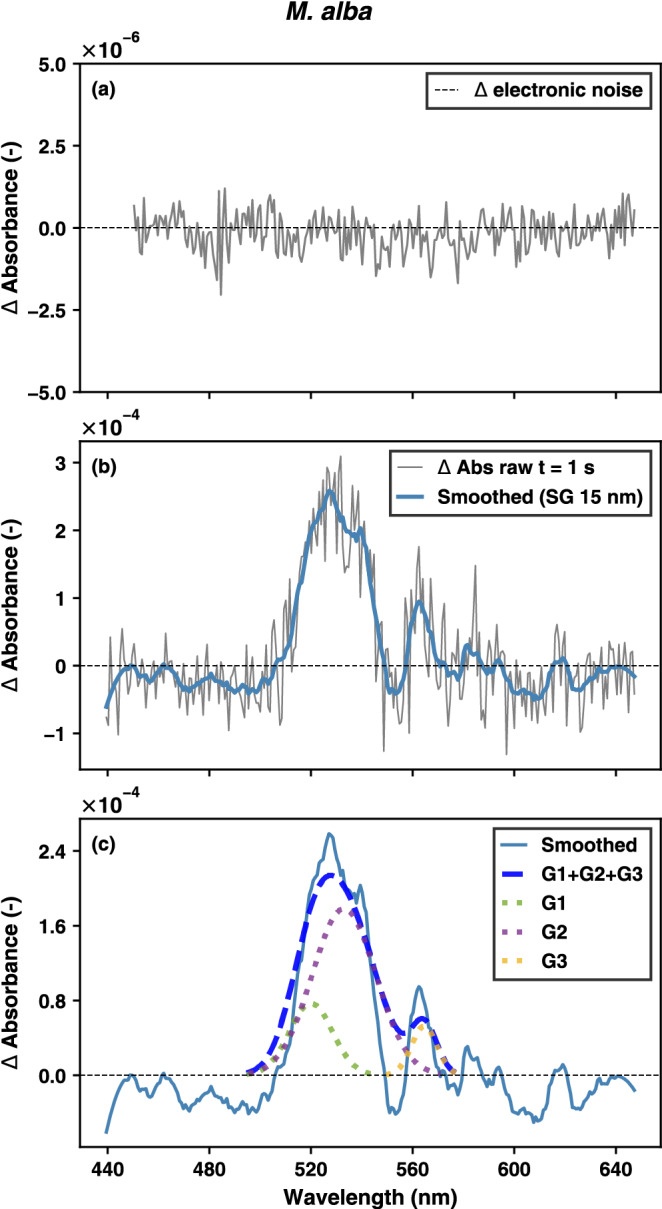
Difference signals in dark (electronic noise, panel **a**) and at the acquisition time of 1 s exposure of 1000 µmol m^−2^ s^−1^ (panel **b**) for *M. alba*. The raw absorbance change spectrum (in grey) is shown together with the corresponding smoothed spectrum (in blue) for illustrative purposes (panel **b**) indicating the underlying signal which is fitted by the three constrained Gaussian components G1, G2 and G3, with means around 520, 535 and 560 nm (panel **c**). The fitting analysis was performed on the raw spectral data

To illustrate the magnitude of spectral changes during the transient and the noise inherent to the measurement, Fig. 2 shows an example of the fitting sequence for *M. alba* at *t* = 1 s (Fig. S1, in the *Supplementary Information* shows the same for the other two species). As illustrated by panel a, the ΔAbs signal is demonstrated to fluctuate randomly around zero under dark conditions, thereby showing that the instrumental electronic noise exhibited no structured spectral features. In contrast, the data presented in panels b–c demonstrate that, under illumination, the raw ΔAbs spectra contain spectral components that are captured by three gaussian functions centered at 520, 535 and 560 nm, respectively. In order to highlight the alterations in the spectrum caused by the dynamics of pigments’ absorptions, a Savitzky–Golay (SG) filter is employed. This filter is a widely utilized technique for the smoothing of hyperspectral noisy signals (Vidal and Amigo 2012). The components that have been fitted show different spectral shapes and temporal evolution following patterns that can be distinguished from spectral fluctuations due to lower magnitude random noise.

### α- and β-carotenoid branch conversions during dark-to-high-light transient

#### Fast transient pigment sampling and HPLC analysis

Leaves of all species were sampled for pigment detection from the same branches used for spectroscopy measurements. Pigment sampling during the dark-to-light transient phase took place every time on two 1 cm diameter leaf disks exposed to identical LED illumination of 1000 μmol m^−2^ s^−1^ as to the spectroradiometer set-up (Fig. 1d). The two dark-adapted leaf disks held by a pair of tweezers were exposed during respectively 1, 2, 3, 5, 7, 10, 15, 20, 30, 40, 60, 90, 120 and 180 seconds, and then immediately frozen in liquid nitrogen (Van Wittenberghe et al. 2021). The same protocol was repeated three times for the full time series, including the sampling of unexposed dark-adapted disks. Samples were stored at −80 °C until further pigment extraction and analysis as described in Rodrigo et al. (2019). Briefly, frozen leaf disks were ground in pure acetone buffered with CaCO_3_ and after centrifugation the supernatant was filtered through a 0.22 µm PTFE filter. The profile of pigment extracts was characterized and quantified by high performance liquid chromatography system (Waters ACQ Arc SysCore HPLC) equipped with a diode array detector (DAD 2998, Waters) set to scan from 300 to 700 nm, and the Empower3 software (Waters®, Barcelona, Spain). A C30 carotenoid column (250 × 4.6 mm, 5 μm) (YMC Europe GMBH, Germany) coupled to a C18 guard column (10 × 4.3 mm, 5 μm) (Teknokroma, Barcelona, Spain) were used with a ternary gradient methanol (MeOH), water and methyl tert-butyl ether (MTBE) (Table S1 in *Supplementary Information*). Each carotenoid (Car) pigment (β-Car, Vio, Ant, Zea, α-Car, Lut, and Lx) and Chl a and b were identified by absorbance spectra and retention time (Fig. S2 and Table S2 in *Supplementary Information*). For each elution, a Maxplot chromatogram was obtained which integrated each pigment peak at its corresponding maximum absorbance wavelength and their contents were calculated using calibration curves of Zea (Extrasynthese), β-Car (Sigma) for α- and β-Car, Lut (Sigma), Ant (CaroteNature), Vio (CaroteNature) for Vio and Neo isomers (Rodrigo et al. 2019, 2015). For Lx quantification, the calibration curve of Lut was used, and the calibration curves of Chl a and b were performed using the corresponding analytical standards (Sigma-Aldrich, USA).

#### Normalization of the dynamic carotenoid trends upon illumination

In order to allow easy and consistent comparison between the different Car dynamics and account for different antenna sizes due to growing conditions and species variability, we normalized for each leaf sample all analysed Car (β-Car, Vio, Ant, Zea, Neo, α-Car, Lut, and Lx) concentration values over the sample’s Chl a molecular pool for graphical visualization and further calculations. Additionally, we focused on the relevance of each pool within the time frame of the transient, considering the variable pool normalized over the total maximum pool. In this way the relevance of each molecule within the regulated photoprotection process is presented in function of its total pool. We define the percentage of the dynamic pool size over the total available pool for each Car as the difference between the maximum (“max”) and minimum (“min”) pool obtained during the sampling performed along the transient time ($\Delta t$) of 3 minutes (see Fast transient pigment sampling and HPLC analysis), normalized by the respective maximal available pool measured along the transient:

6 $$Dynamic\,Car\,pool\left[ \% \right] = {{\left[ {{{Car} \over {Chla}}} \right]_{\Delta t}^{max} - \left[ {{{Car} \over {Chla}}} \right]_{\Delta t}^{min}} \over {\left[ {{{Car} \over {Chla}}} \right]_{\Delta t}^{max}}} \times 100\% $$

To further explore the dynamics of each Car during the transition to high light and compare among each other, we calculated the variation in pigment content at each sampling time point ${t_i}$ relative to its initial value in dark-adapted state (${t_0}$). This difference was normalized over the maximum variable pool to pronounce the trend of the dynamic fraction of each Car, following the formula:

7 $$Normalized\;Car\;at\;{t_i}\left[ - \right] = \frac{{{{\left[ {\frac{{Car}}{{Chla}}} \right]}_{{t_i}}} - {{\left[ {\frac{{Car}}{{Chla}}} \right]}_{{t_0}}}}}{{\left[ {\frac{{Car}}{{Chla}}} \right]_{\Delta t}^{max} - \left[ {\frac{{Car}}{{Chla}}} \right]_{\Delta t}^{min}}}$$

Lastly, we evaluated de novo synthesis of pigment pools at two pool levels. First, we evaluated de novo synthesis of the VAZ and LxL xanthophyll cycle pools from their respective precursors β-Car and α-Car by analyzing the statistical changes of the [Vio+Ant+Zea] and [Lx+L] pools along the transient, with respect to the dark-state pool. Next, we evaluated if de novo synthesis took place for the entire β- and α-branches, from their respective precursor lycopene, by analyzing the transient changes in [Vio+Ant+Zea+Neo+β-Car] and [Lx+L+α-Car].

#### Statistical analyses

Statistical analysis of pigment data was performed using the software IBM SPSS Statistics (v.26.0). To compare between pigment concentration (normalized over Chl a) at each time point and pigment content in darkness for each species and growing light condition, we used Student’s t-test. Normality and homoscedasticity of data was assessed using a Shapiro–Wilk’s and a Levene’s test, respectively. To evaluate the relationship between the quenching parameter and pigment composition, we calculated Pearson’s correlation coefficient and its associated *p*-value for each experimental group in MATLAB. The quenching parameter was derived from fluorescence measurements, while the pigment composition consisted in Vio or the sum of the specific carotenoids which significantly increased at *t* = 180 s for each species and light condition, in all cases normalized to Chl a. Both datasets were interpolated to a common time axis to ensure alignment, and only time points with valid data for both quenching and pigment composition were included in the analysis. In all cases, *p* < 0.05 values were considered statistically significant (*). *p* values below that were represented with two (*p* < 0.01) or three (*p* < 0.001) asterisks.

## Results

### Dynamic photoprotective carotenoid pools

All the measured xanthophylls and carotenes demonstrated dynamic chemical conversions within the dark-to-high-light transition time of 180 seconds indicating their role in the quick photoprotection (Table 1). The proportion of the dynamic pool over the maximum pool ranged between 10 and 100%, considering the three species and sun-versus-shade growing conditions. As expected, the pigments of the VAZ pool showed a very high activation response upon the light transient (up to 100%). High dynamic pool proportions were observed for Zea across the three different species and light conditions (77–100%), indicating that Zea was almost absent at darkness and the entire pool was formed in response to illumination. Only in the case of *M. alba*’s sun-grown leaves the portion of Zea’s dynamic pool was lower (50%). Ant, often considered only an intermediate molecule in the VAZ cycle, showed pronounced dynamics in all species (70–97%), with shade-grown leaves of *Q. ilex* showing the highest dynamical pool (97%). Neo showed a lower dynamic pool upon illumination (11–41%), in which shade-grown leaves consistently showed higher dynamic changes compared to sun-grown leaves across all species, suggesting a greater role for Neo in photoprotection under low-light conditions. Also, β-Car presents a non-negligible dynamic chemical response under sudden light, with dynamic proportions up to 68% in shade grown leaves of *M. alba*. In the case of α-Car, a variable light-driven dynamic pool was found, ranging from 37% (*Q. ilex*, shade) up to 100% (*M. alba*, sun).

**Table 1:** Mean percentage of light-dynamic pigment pools (%) (± standard error) for the carotenes and xanthophylls, normalized over the Chl a pool (for inter-leaf comparison) calculated from Eq. 6, using maximum and minimum pool concentrations (mol mol^−1^) sampled during Δ *t* = 180 s upon a sudden light exposure (*n* = 3)

(max-min)/max * 100%	*C. australis*		*M. alba*		*Q. ilex*	
	Shade	Sun	Shade	Sun	Shade	Sun
[Neo/Chl a]	41 ± 14	32 ± 4	22 ± 2	14 ± 1	29 ± 12	11 ± 1
[Vio/Chl a]	66 ± 15	61 ± 3	70 ± 2	51 ± 4	80 ± 4	44 ± 2
[Ant/Chl a]	70 ± 7	91 ± 5	70 ± 3	71 ± 5	97 ± 0	80 ± 1
[Zea/Chl a]	100 ± 0	99 ± 1	96 ± 1	50 ± 2	100 ± 0	77 ± 2
[β-Car/Chl a]	31 ± 4	30 ± 3	68 ± 15	26 ± 3	43 ± 1	16 ± 2
[α-Car/Chl a]	72 ± 12	79 ± 2	98 ± 1	100 ± 0	37 ± 8	56 ± 7
[Lut/Chl a]	15 ± 4	10 ± 2	29 ± 2	17 ± 2	15 ± 3	10 ± 1
[Lx/Chl a]	100 ± 0	nd	92 ± 1	58 ± 1	79 ± 1	46 ± 3

Lutein also shows a light-dynamic pool in the order of 10–29% across all the species measured. Hence, despite a lower relative importance compared to the VAZ pools, a clear and significant role in the light-dynamic response seems to be carried by the Lut molecules. In contrast, Lx, despite having a very small absolute pool size in darkness (Table S3 in *Supplementary Information*), a very high dynamical percentage is observed in the quick photoprotective response, particularly in the case of shade-grown leaves (79–100%). Sun-grown leaves overall showed a lower proportion of dynamic pools of various xanthophylls across the three species, which could be explained by having already developed pigment pools, with higher concentration of those pigments before illumination (Table S3 in *Supplementary Information*).

Although the overall trends in the proportion of the Car dynamics are rather similar, small species-specific differences can be observed. For instance, *C. australis* exhibited the highest overall changes in β-branch pigment pools, suggesting a strong reliance on VAZ cycle-mediated photoprotection in this species. In contrast, *M. alba* showed the most moderate changes in the VAZ pool, but the highest changes in the LxL cycle pool and α-Car and β-Car. *Q. ilex,* for its part, exhibited and intermediate response for the VAZ pool, and the lowest LxL cycle and carotenes pools changes.

### Chaotic early trends in balancing dynamic carotenoid pools

**Fig. 3:**
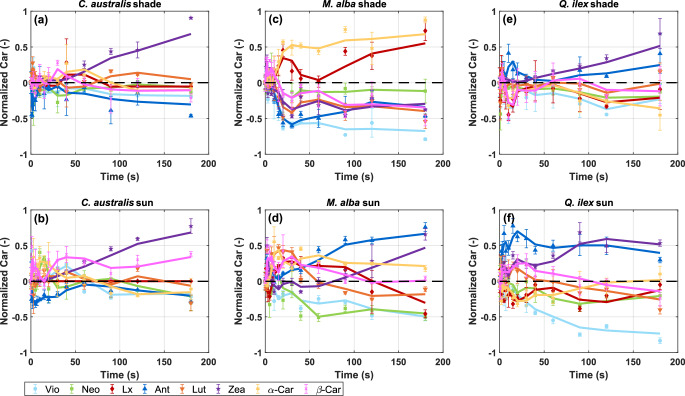
Temporal dynamics of individual carotenoids during 180 s of high light exposure in shade- (panels **a**,**c**,**e**) and sun-adapted (panels **b**,**d**,**f**) leaves of *C. australis*, *M. alba* and *Q. ilex*. Changes in carotenoid composition relative to dark-adapted samples were calculated from Eq. 7, and mean values ± standard error are shown in each panel (*n* = 3)

**Fig. 4:**
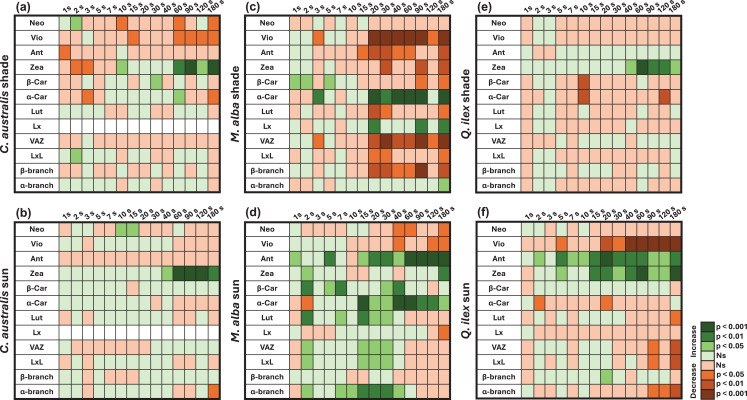
Significance plot for the evolution of the normalized (over Chl a) carotenoid pools relative to dark-adapted samples, as shown in Fig. 3. Panels show the results for shade- (panels **a**,**c**,**e**) and sun-adapted (panels **b**,**d**,**f**) leaves of *C. australis*, *M. alba* and *Q. ilex*. The significance of increases (green) and decreases (orange) in each pigment pool are evaluated by comparison with its pool in darkness (*n* = 3). Statistical differences were evaluated using a Student’s t test with *p*-values > 0.05 considered as non-significant (ns). Blank squares mean that the measurement was not within the detection limit

To observe when the strongest fluctuations in carotenoids dynamics take place, the temporal trend in normalized pigment pool point relative to its initial value in darkness (Eq. 7) is presented across the three studied species under both sun-grown and shade-grown conditions (Fig. 3). Further, statistical differences are shown for the different pigment pools (normalized to Chl a) along the transient, in comparison to the dark-adapted state (Fig. 4).

Overall, all pigments show very quick dynamics within seconds upon the start of the light transient (Fig. 3). The behaviour of most pigments is observed as irregular (or even chaotic) within these first seconds, often leading to both increases and decreases, with respect to the dark-state (*t* = 0 s) pools (Figs. 3 and 4). It demonstrates the strong non-steady-state behaviour of the antenna composition during the transition of a dark to the light-adapted state and the interplay of both α and β-branch carotenoids.

Quick pool increases are commonly seen for Ant, Zea, and Lut (Figs. 3 and 4). In some cases, these pool increases are found significant within the first couple of seconds, e.g., for *Q. ilex* sun (Ant: *p* < 0.05), *M. alba* sun (Ant: *p* < 0.05, Zea: *p* < 0.05), *M. alba* shade (β-Car: *p* < 0.05) and *C. australis* shade (Neo < 0.05) (Fig. 4). In all cases, significant pool changes are commonly recorded within the first 20 seconds upon the light transient. Zea shows a rapid increase upon illumination, which sustains until the end of the transient (180 s), confirming its well-documented role as the primary component of the VAZ cycle in photoprotection (Figs. 3 and 4). Interestingly, strong differences are observed in the steady-state pigment pools between sun and shade-grown *M. alba* leaves. At the end of the recorded transient (*t* = 180 s) sun-grown leaves have significantly increased Ant (*p* < 0.001), Zea (*p* < 0.01) and α-Car (*p* < 0.05) pools, and shade-grown leaves have only significantly increased Lx (*p* < 0.01) and α-Car (*p* < 0.001) pools, with respect to dark-state pools (Fig. 4c,d).

Ant also exhibits a marked and rapid increase across all species, and remains with a significantly increased pool at *t* = 180 s of the sun-grown leaves of *M. alba* and *Q. ilex* (Fig. 4d,f) reinforcing the idea that it actively participates in short-term photoprotection rather than merely serving as a transient intermediate in the VAZ cycle (Goss et al. 1998). Vio shows a sharp initial decline (Fig. 3) and is significantly reduced at *t* = 180 s for all species (Fig. 4), consistent with its role as a precursor in the VAZ cycle. The extent of its depletion varies, being most pronounced in shade-grown leaves, where a greater proportion of the available pool is converted to downstream photoprotective xanthophylls. Notably, in *C. australis* (both shade- and sun-grown), Vio is partially retained throughout the time course (Fig. 3a,b), suggesting a modulation of its photoprotective role. Neo displays a more gradual and less pronounced decline, reinforcing the idea that its involvement in photoprotection is comparatively minor relative to Vio or Zea (Figs. 3 and 4). Lx exhibits a sharp increase in *M. alba* shade leaves (Fig. 4c, *t* = 20 s), agreeing with its high dynamic pool found for this species and growing condition (Table 1). However, pools of Lx at *t* = 180 s do not appear significantly increased in the rest of the cases (Figs. 3 and 4) and Lut remains relatively stable across most conditions, though slight increases are observed in some cases, particularly in shade-grown *M. alba* (from 2 to 30 s) (Fig. 4a,c). This suggests that although LxL cycle’s dynamics are more limited than those of the VAZ cycle pigments, it may still participate in photoprotective responses, especially during the first seconds of illumination and before other pigment increases take over.

β-Car and α-Car also showed oscillations in their pools upon illumination in all species and light conditions (Fig. 3). In the case of β-Car, its pool was significantly decreased at *t* = 180 s in the case of *M. alba* shade leaves (Fig. 4c). In other cases, it showed strong oscillations before *t* = 180 s, with significant increases (e.g., *M. alba* shade leaves *t* = 1, 2 and 5 s, *M. alba* sun leaves *t* = 2–40 s) and decreases (*Q. ilex* shade leaves *t* = 10 s, *M. alba* shade leaves *t* = 120 s) depending on the species and light condition (Fig. 4). For its part, α-Car was significantly decreased at *t* = 180 s in *C. australis* shade leaves (Fig. 4a) and also showed significant decreases for *Q. ilex* sun and shade leaves (Fig. 4e,f). However, in the case of *M. alba*, α-Car was significantly increased at several time-points for both shade and sun leaves, even at *t* = 180 s (Fig. 4c,d). These observations indicate that β-Car and α-Car pools also vary depending on species and growing conditions.

These early-second dynamics in pigment pool profiles reveal that, despite the apparent long-term stability of certain pigments like Lut, all pigments exhibit notable fluctuations during this early phase. This highlights that even seemingly static pigments undergo rapid adjustments in response to high light before reaching a more stable state. While the extent and functional significance of these changes differ among pigments, the observed dynamics suggest that multiple pigment pools are responsive during the initial phase of photoprotective adjustment.

### Oscillating mirror trends show balancing dynamic carotenoid pools

**Fig. 5:**
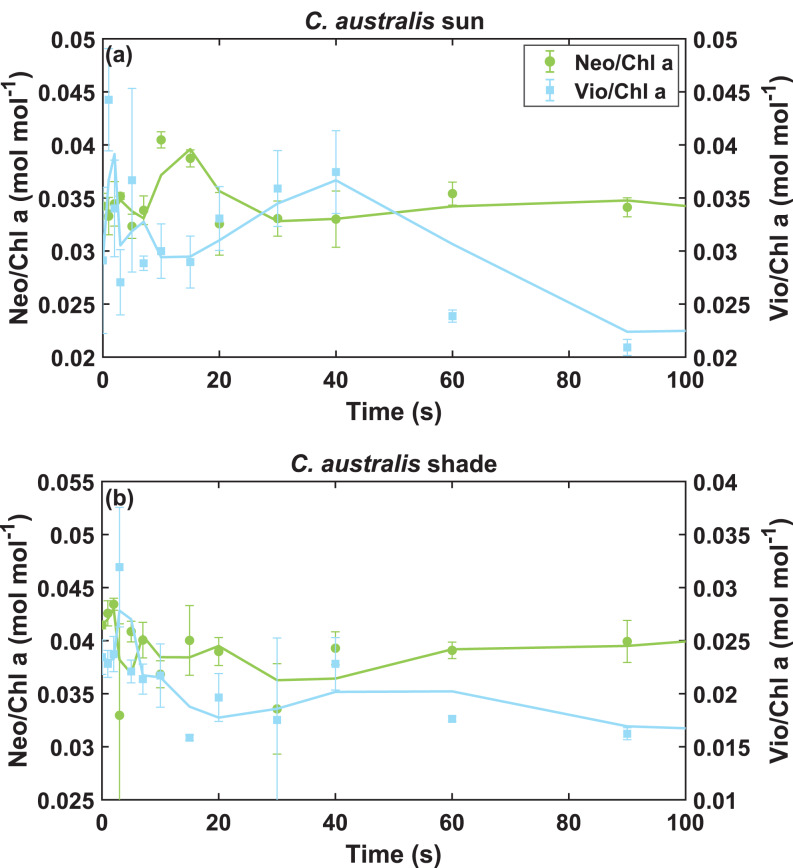
Xanthophyll trends in the first 100 s of illumination indicating the quick oscillating dynamics of Neo complementing the dynamics of Vio for sun-adapted (panel **a**) and shade-adapted (panel **b**) leaves of *C. australis*. Data shown are the mean pigment concentration (normalized over Chl a, mol mol^−1^, *n* = 3) ± standard error of Neo and Vio showing both left and right axes in the same difference range and the 2-period moving mean trendline

When analysing early pool conversions and their correlation with F decrease (Fig. S3 in *Supplementary Information*), a sudden (and unexpected) increase in [Vio/Chl a] seemed to cause a mismatch in the correlation around *t* = 20–40 s in some of the cases. This increase seen as a bump in [Vio/Chl a] can be clearly observed in *C. australis* sun leaves. Further analysis of this anomaly revealed an opposite trend in Neo levels, when plotting both in the same difference range in mol mol^−1^ (Fig. 5), indicating that Neo pool can be directly or indirectly linked to the increase of Vio during this phase. This oscillating mirror trend was less strong in most of the other cases, where stronger Vio de-epoxidation took place (e.g., Fig. 3c).

**Fig. 6:**
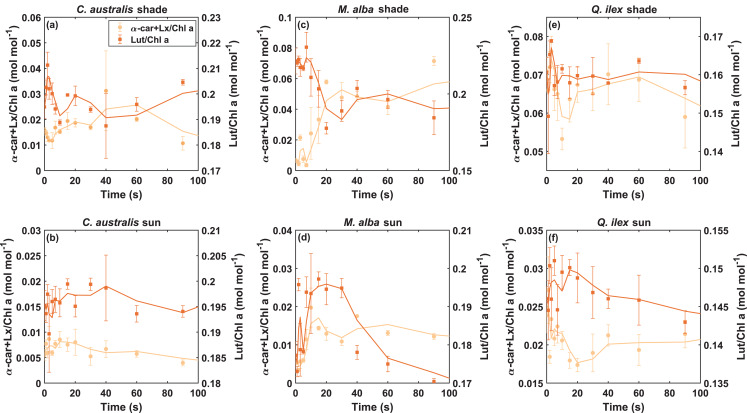
Quick oscillating dynamics of α-Car+Lx and Lut pools during the first 100 s of high light exposure in shade- (panels **a**,**c**,**e**) and sun-adapted (panels **b**,**d**,**f**) leaves of *C. australis*, *M. alba* and *Q. ilex*. Data shown are the mean pigment concentration (normalized over Chl a, mol mol^−1^, *n* = 3) ± standard error with a 2-period moving mean trendline. Left and right axes are in the same difference range to allow easy comparison of molecular conversion dynamics

Other clear examples of quick oscillating conversion dynamics were observed in the α-branch, showing the activation of the LxL cycle within less than 20 seconds for several cases (Fig. 6).

A very quick increase in Lut pool matched with the sum of the depleted Lx and α-Car pools in the case of *C. australis* (shade), *M. alba* (sun) and *Q. ilex* (sun), showing consistent molecular changes (Fig. 6a,d,f). These observations demonstrate the very quick activation of the LxL cycle, involving also α-Car responses. Quick activation of Lut pools is however often followed by an immediate or steady decrease after 20 s of light exposure.

These observations prove that, while the overall trends in pigment dynamics provide insight into their photoprotective roles, a closer look at the very first seconds of illumination reveals a more intricate and highly responsive interplay between different xanthophylls and carotenes. Rather than a steady linear response, pigments undergo rapid interconversions, often oscillating before “stabilizing” towards an equilibrium. This transient behaviour highlights the very quick non-equilibrium nature of pigment dynamics during the initial adaptation to high light.

### Quick de novo synthesis of carotenoids

**Fig. 7:**
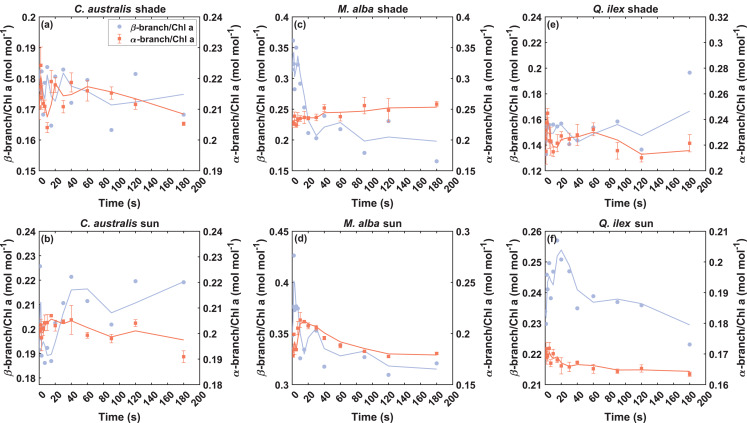
Transient trends of total α-branch (α-Car+Lut+Lx, orange, left y-axis) and β-branch (β-Car+Vio+Ant+Zea+Neo, purple, right y-axis) pools during 180 s of high light illumination in shade (panels **a**,**c**,**e**) and sun-adapted (panels **b**,**d**,**f**) leaves of *C. australis*, *M. alba* and *Q. ilex*. Data shown are the mean pigment concentration (normalized over Chl a, mol mol^−1^, *n* = 3) ± standard error, and the trend line shows the 2-period moving mean. Statistics of the pools with respect to the dark-adapted pools are shown in Fig. 3

Quick trends in the overall α- and β-branch pools further revealed that de novo synthesis of carotenoids took place at the whole branch level (Fig. 7). Even though the overall changes were not always statistically significant from the dark-adapted state (Fig. 4), trends showed a quick activation or depletion of the entire branch pools within the first 20–30 seconds (Fig. 7), which could be also observed in the α-Car and β-Car pools (Fig. S4 in *Supplementary Information*). Overall, this points toward possible rapid carotenoid biosynthesis from the α- and β-Car precursor, contributing to the replenishment of xanthophylls required for photoprotection as a first response within the first 10–20 s. Despite the fact that the β-branch shows a larger dynamical range (mol mol^−1^ Chl a) in most of the cases, the activation response of the α-branch seemed to occur faster in some of the cases (e.g. Fig. 7b,f). Interestingly, pigment trends for *M. alba* shade leaves show that the α-branch of the xanthophylls seems to be more involved in the response to high light transient illumination, with significant increases in its α-branch pigments at *t* = 180 s, in contrast to the significantly decreased β-branch pigments (Figs. 3c and 4c). The opposite patterns in α- and β-branch pools (Fig. 7), which might be mostly appreciated from the sun leaves in general, may reflect a shift in precursor allocation between the α- and β-branches in response to light stress, potentially optimizing the downstream synthesis of distinct xanthophylls involved in photoprotection. Notably, the extent of de novo synthesis appears to vary by species and light environment, which also could implicate the differences in available dark-state pools (Table S3 in *Supplementary Information*).

### Early-transient fluorescence and absorbance changes

**Fig. 8:**
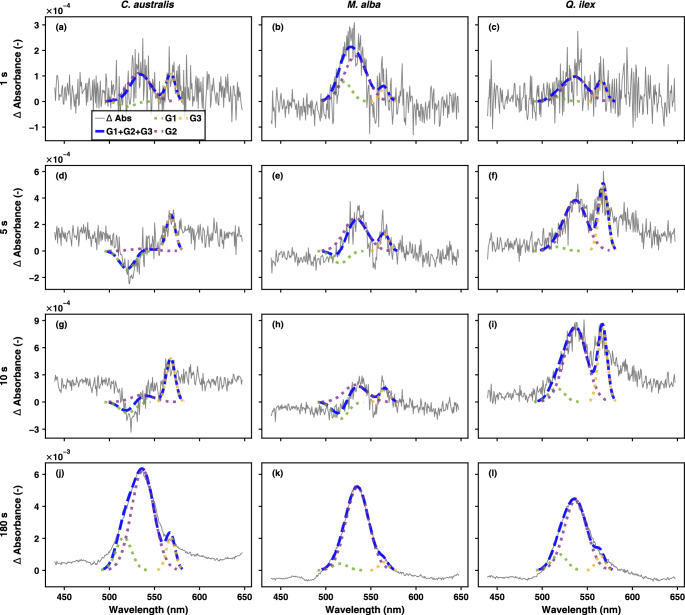
Transient absorption spectra upon light exposure of 1 s (panels **a**–**c**), 5 s (panels **d**–**f**), 10 s (panels **g**–**i**) and 180 s (panels **j**-**l**) are shown for sun-grown leaves of respectively *C. australis*, *M. alba* and *Q. ilex*. Raw, unsmoothed difference spectra are shown. The measured absorbance spectra are modeled through 3 Gaussians G1, G2 and G3 with means around 520, 535 and 560 nm

**Fig. 9:**
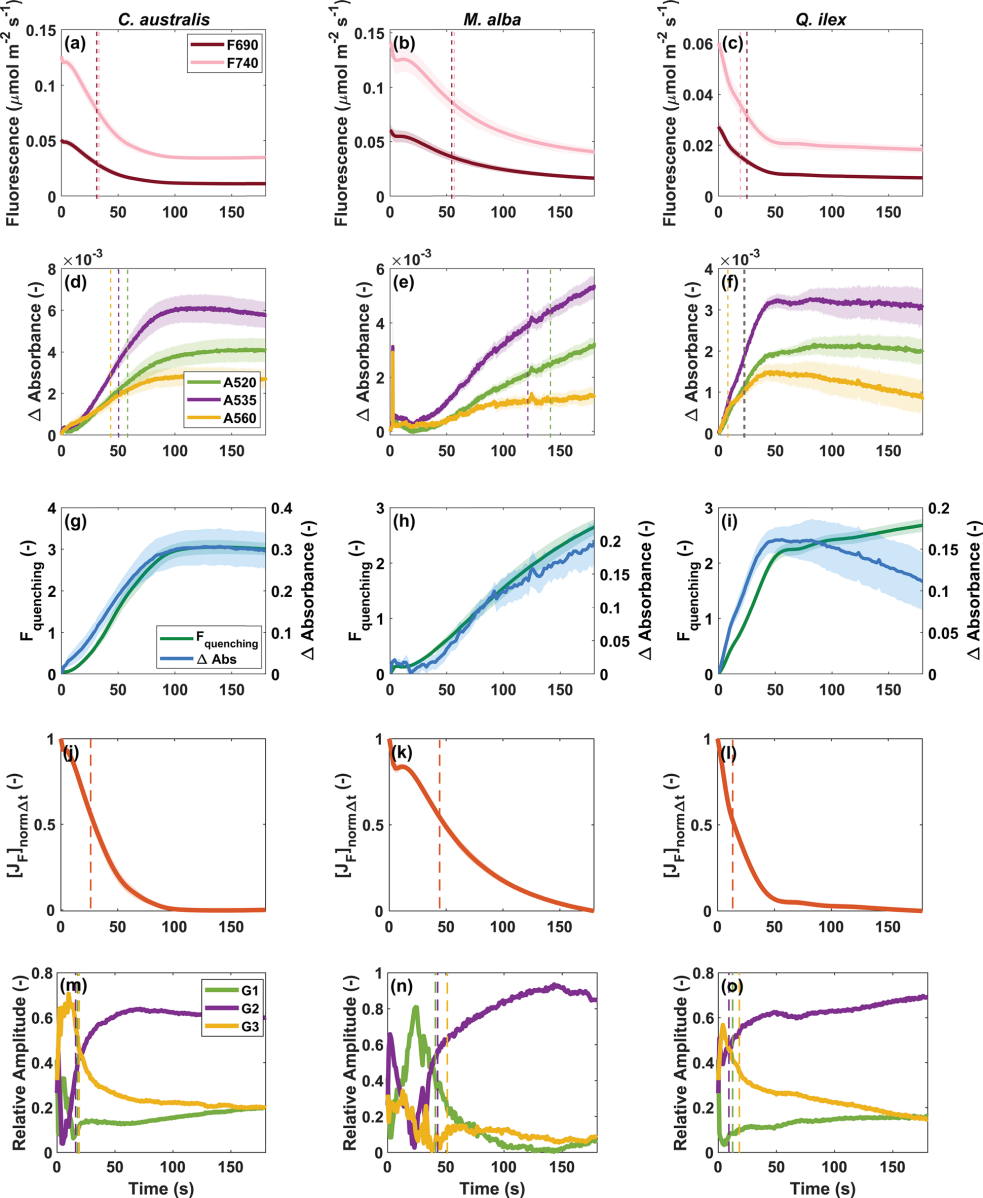
Analysis of fluorescence and absorbance kinetics during dark-to-high-light transients in sun-adapted leaves (*n* = 6) of *C. australis*, *M. alba*, and *Q. ilex*. Decrease of fluorescence peaks (F690, F740) (panels **a**–**c**) and increase of Δ absorbance peaks (A520, A535, A560) (panels **d**–**f**) are shown (mean ± standard error). Panels **g**–**i** show the F quenching calculated based on the total fluorescence flux (J_F_) from the emission in 655–850 nm (Eq. 2), together with the total Δ absorbance spectrally integrated (500–600 nm). Panels **j**–**l** show the normalized total fluorescence decay over time (Eq. 3). To illustrate distinct kinetics, vertical dashed lines show the half-time of fluorescence and Δ absorbance corresponding to each wavelength during the transient time. Panels m-o show the relative amplitude of each Gaussian during the transient time of 180 s from the measured absorbance spectra modeled in Figs. 7 through 3 Gaussians G1, G2 and G3 with means around 520, 535 and 560 nm. For each relative amplitude the half-time to reach its maximum or minimum is indicated as vertical dashed line

To understand how quick these pigment pools contribute to the antenna state behaviour, we examined the transient changes in both the absorbance and fluorescence spectra (Figs. 8 and 9). Overall, the decreasing fluorescence flux (*J*_F_) response upon high light exposure follows a biphasic pattern, with an initial rapid decline in fluorescence occurring within the first 20 seconds, followed by a slower phase that stabilizes around 100–120 seconds (Fig. S3 in *Supplementary Information*). The fluorescence peak ratio also showed very quick variation along the transient, showing an overall decreasing trend, except in some cases of *M. alba* (Fig. S5 in *Supplementary Information*). These F690/F740 trends may indicate quick changes in the Chl absorbance behaviour (which cannot be shown due to the cut-off filter). Absorbance changes in the green region appear within the first seconds (Fig. 8a–i), as in the same range that carotenoid pool dynamics were noticed (Fig. 4). These very early absorbance changes within the 500–600 nm range are modelled using fixed Gaussian shapes with means around 520, 535 and 560 nm. Both positive and negative amplitude weights were fitted in the early seconds, becoming eventually dominated by positive absorbance changes at *t* = 180 s (Fig. 8j–l). Note that we do not fit the noise levels, but the signal which is superimposed in the difference spectra (see comparison with smoothed difference spectra in Fig. 2 and Fig. S1 in *Supplementary Information*). Broader overlapping spectral broadening effects were not fitted, which caused a sort of baseline discrepancy, noticeable in the early seconds. The NRMSE of the fitting results are shown in Fig. S6 in *Supplementary Information*. Further, the Chl a F decrease showed a faster decline compared to the absolute absorbance changes, analysed from the 520, 535 and 560 nm peaks (Fig. 9a–c vs. Fig. 9d–f). This can also be observed from the half-times indicated for both the fluorescence and absorbance changes (Fig. 9a–f). Interestingly, when expressing the F decrease through the NPQ-equivalent formula, named F quenching (Eq. 2), a consistent match was obtained with the overall absorbance increase (Fig. 9g–i). Hence, this NPQ-equivalent parameter highlights the relative changes in F showing a slower trend compared to the quick overall fluorescence decline. Fluorescence decline is shown as the normalized total fluorescence flux decay to facilitate comparison between the different cases (Fig. 9j–l). In addition, we show the relative amplitude weights of the fitted gaussians, with the sum of the three relative weighted amplitudes being equal to 1 (Fig. 9m–o). The dynamics of these relative weights show an altering behaviour of their fitting importance within the first 10–40 s. Remarkably, the half-time of the relative weight of the dominant Gaussian (i.e., G2 at 535 nm) to reach its maximum coincides with the half-time of the normalized F decrease. Eventually, at *t* = 180 s, the Gaussian’s relative weights are quite similar across the different species with a dominant Gaussian at 535 nm (Fig. 9m–o).

With the aim to find a coinciding trend between the chemical changes and F quenching, we first aimed to correlate the F dynamic profile with the Xan pools. At first sight, the early transient phase (0–30 s) shows highly heterogeneous carotenoid dynamics (Figs. 3, 4 and 5), making it difficult to disentangle the potential causality of individual pigment species. Instead, the full transient time until 180 s was considered, in which the [Vio/Chl a] decrease shows a significant (*p* < 0.05) temporal correlative effect with F decline for most species and light conditions (Fig. S3 in *Supplementary Information*). These results also indicate that Vio de-epoxidation generally occurs promptly, within the first 10 s, during the early F decline (Fig. S3 in *Supplementary Information*). We further analysed the correlation between fluorescence decrease and the pigments that showed a significant net increases at *t* = 180 s (Fig. S7 in *Supplementary Information*). A clear trend was observed: pigments that accumulated significantly by the end of the transient—such as Zea, Ant, Lx or α-Car in specific species and light conditions—were associated with the second, slower phase of fluorescence decrease.

**Fig. 10:**
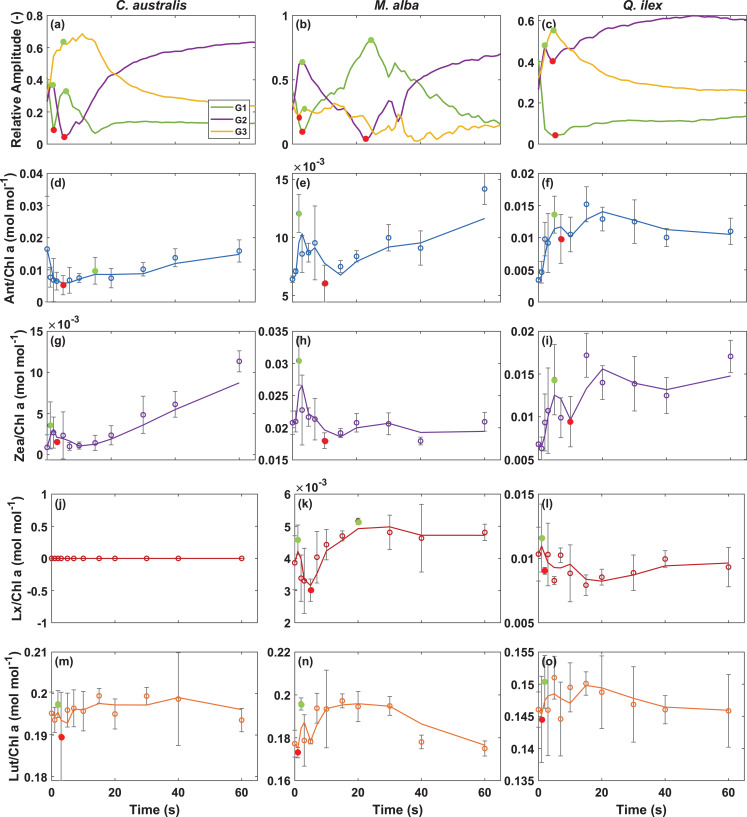
Relative amplitude of the three Gaussian components (G1, G2, and G3) obtained from deconvolution of the measured absorbance spectra shown in Fig. 8 over a transient period of 60 s (panels **a**–**c**). The spectra were modeled using three Gaussian functions with mean wavelengths centered at approximately 520 nm (G1), 535 nm (G2), and 560 nm (G3). Individual xanthophyll cycle pigment levels during high light exposure in sun-grown leaves of *C. australis*, *M. alba* and *Q. ilex* (panels d-o, *n* = 3). (**d**–**f**) Antheraxanthin (Ant/Chl a), (**g**–**i**) zeaxanthin (Zea/Chl a), (**j**–**l**) lutein epoxide (Lx/Chl a) and (**m-o**) lutein (Lut/Ch a) content normalized to chlorophyll a are shown for each species. Error bars represent ± standard error and trend lines represent a 2-period moving mean. In all panels dots indicate the first detected relative extreme during the transient response, with maxima highlighted in green and minima in red

We further illustrated the importance of the fitted Gaussians with respect to the very quick dynamics of the main xanthophyll candidates for quenching (Fig. 10). For the transient time of the first 60 s the relative amplitudes of G1, G2 and G3 are shown, indicating the first minima and maxima observed (Fig. 10a–c). Similarly, the minima and maxima were highlighted for Ant, Zea, Lx and Lut pigments. Those minima and maxima seem to match quite well the underlying Gaussian behaviour (Fig. 10d–o). For example, in the case of *M. alba*, Ant and Zea reach a first quick maximum at *t* = 2 s (Fig. 10e,h), like the time as when the relative amplitude of the G2 peak reaches a first maximum in the fitting (Fig. 10b), whereas both trends show a decrease afterwards, until *t* = 20–30 s, to further increase again, in the case of Ant (Fig. 10e). In the case of *Q. ilex*, the moving trends in both the Gaussian amplitudes and the pigments occur even faster. Ant and Zea show a quick maximum around *t* = 5 s, followed by a decrease to a first minimum at *t* = 7–10 s (Fig. 10f,i). This trend can also be observed by the relative amplitude of the dominant Gaussian G2 (Fig. 10c).

## Discussion

### Very fast Xan/Chl antenna changes triggering EET followed by regulated heat dissipation

The photoprotective role of xanthophylls during the transition from dark to high-light conditions starts with a complex interplay of dynamic pigment conversions, both in the α- and β-branches. Our results demonstrate that during the prompt decrease of fluorescence upon a light transient, multiple chemical conversions occur involving the VAZ cycle, the LxL cycle, and their carotene precursors. These early conversions take place within the first seconds, reaching pigment pool maxima within 10 to 20 s upon light exposure, which are found significant in several cases (Figs. 3 and 4). Significant absorbance changes at 520 or 535 nm, used as an indirect proxy of the build-up of the proton gradient (Allorent et al. 2018) or the Xan-related conformational changes (Bilger et al. 1989; Ilioaia et al. 2011; Peguero-Pina et al. 2013), are often seen slower (Figs. 10 and S8 in *Supplementary Information*). These findings suggest that the activation of VDE and the onset of the chemical conversions within the xanthophyll cycle occur very rapidly after illumination, potentially in parallel with early phases of proton-gradient formation. Hence, while protonation and a built-up electric field may be an essential trigger for additional slower processes such as the conformational changes and the activation of regulated heat dissipation, our results indicate that some of the initial chemical changes proceed on a faster timescale, shown by pigment analysis (Figs. 3 and 4) and the early Gaussian-characterized changes in the absorption profile (Figs. 8 and 10). Hence, it could be that a lower level of lumen acidification is sufficient to activate VDE and the xanthophyll cycle at the very early onset of illumination exposure. Under darkness, VDE activation has been also observed (Fernández-Marín et al. 2009), which the authors propose could have happened due to a decrease in chloroplastic pH during drought or due to the association of VDE activity with special lipidic structures formed during dehydration in the thylakoid membranes.

Interestingly, the observed stoichiometric modifications co-occur with the early absorbance components, exposing both negative and positive features (Fig. 8a–i). Such spectral features have been also ascribed to electrochromic absorption shifts of carotenoids, in the same (or faster) timescale (Kramer et al. 1999; Viola et al. 2019), or red shoulders of distinct Xan S0–S2 absorption contributions to triplet-minus-singlet absorption (T-S) spectra (Peterman et al. 1997). While electrochromic changes in the antenna are expected to be at play, we even further propose these changes can be (partly) attributed to true absorbance changes upon the chemical conversions. These conversion dynamics can be observed through the kinetics of the underlying absorbance components within the first 10 s, modelled as an interplay of different Gaussians which show quick relative alterations upon amplitude normalization (Fig. 10). Based on the similarity in the quick conversion kinetics, Ant or Zea are considered the best candidates for a role in the central Gaussian (G2) at 535 nm (Fig. 10). Earlier work came to the same interpretation using a similar Gaussian fitting approach for *M. alba*, *Fagus sylvatica* and *Juglans regia* leaves (Van Wittenberghe et al. 2021).

**Fig. 11:**
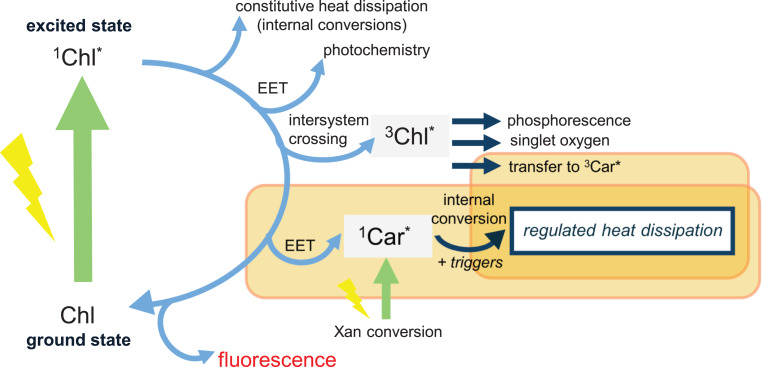
Radiative and non-radiative transitions of a singlet excited Chl a molecule. Radiative transitions are represented by fluorescence, and non-radiative transitions are represented by internal conversions (constitutive heat dissipation), intersystem crossing leading to triplet excited Chl (^3^Chl*), and excited energy transfer (EET) towards either photochemistry or a single excited Car (^1^Car*) quencher molecule. Note that triplet Car states (^3^Car*) have been also reported as efficient quenchers of ^3^Chl*. EET towards a newly formed Car quencher molecule from the xanthophyll (Xan) cycle and the further process of internal energy quenching through a particular excited Car molecule (^1^Car* or ^3^Car*?) can be observed from in vivo spectroscopy using dark-to-high-light transients

Such fast altering of the antenna composition, favouring xanthophyll pools with a short lifetime, should therefore be considered as the fast excitation energy transfer (EET) quenching path, prior to the activation of regulated heat dissipation requiring additional triggers (Fig. 11). The non-steady state behaviour in Xan/Chl stoichiometry has however been rarely brought to the attention as a cause for (partial) fluorescence decline in such short timescale due to the lack of high temporal resolution measurements, and due to the absence of EET quenching to new quencher molecules in the commonly used formulas from PAM fluorimetry measurements. Information on non-photochemical quenching has been classically obtained through the use of saturating flashes being considered as critical for separating the photochemical quenching versus the non-photochemical effects, following the two-state model with the RC of PSII being either in a quenched or unquenched state (Stirbet and Govindjee 2012). Recently, the dogma of this two-state model and the conventional parameters based on this model are argued to have lost their physical meanings (Garab 2025). We can argue also for this based on our observations.

It is shown that both normalized F decline and the “F quenching” (NPQ equivalent) partly coincide with the trends in absorbance:

1. The normalized F decline shows a similar half-time as the half-time of the relative amplitude of the dominant Gaussian (G2 at 535 nm), demonstrating simultaneous trends in the early 20–30 seconds of the transient (Fig. 9j–o), and
2. The “F quenching parameter” (NPQ equivalent), highlighting a relative change of the F decline through the formula (F-F’)/F’ (normalized difference) shows a similar half-time as the half-time of the amplitude of a dominant Gaussian-modelled absorbance (here, G2 at 535 nm), demonstrating simultaneous trends over the full transient (Fig. 9g–i).

This has two major consequences. The first consequence is that fluorescence dynamics are both in the first order as in the normalized difference form quite well aligned with the kinetics in the absorbance due to specific pigment broadening. Although the second, NPQ-like, aligned trend should be rather considered an indirect result of the F declining trend, the quick first trend points to a change in the absorbance upon prompt F decline and Xan formation. This suggests that, apart from variable dynamic photochemical quenching (i.e. different reaction in PSI vs. PSII RC centers), EET to new xanthophylls can play a relevant role in this quick phase of F decline.

A second consequence is that both the quick (second-scale) and slower absorbance trends can be characterized by partly similar Gaussians (with possible negative features due to molecules being converted), suggesting co-depending mechanisms. It must be pointed out that the current fitting approach forces the fitting of (only) three fixed Gaussian positions and widths. While there may be more complexity in the amount and position of peaks in the quick conversion phase, it is also clear that during the slower quenching phase only one dominant Gaussian is sufficient, whereas the other two probably fit the remaining noise (e.g., Fig. 8j–l). Hence, not all peaks that appear in the early phase, may appear or be relevant in the slower phase. It is further important to note on the one hand that the slower absorbance increases may depend on external triggers and do not per se have a direct impact on the F quenching as they do not coincide with the F decline itself (Fig. 11). But absorbance peaks that occur in both processes, which is clearly the case for the 535-nm peak, can however point to the strongly allowed S0–S2 electronic transition of a particular, red-shifted xanthophyll molecule in the EET process, being further triggered in the next step. This suggested hypothesis on a common 535-nm peak due to both electronic transition and de-excitation of electrons, leads to the plausible occurrence of vibrationally hot S0 states (rather than a separate state) as the latter quenching mechanism. Whether the dynamic Chl Qy band plays an additional role in the short-term energy quenching could not be verified in this study, being only dedicated to the 500–600 nm spectral range. However, from other in vivo studies we observed additional Chl peaks at 615 and 645 nm from the leaf scale (Van Wittenberghe et al. 2019) and a Chl Qy band peak at 695 nm from the canopy scale (García-Martínez et al. 2025).

### Individual Xan behaviour in fluorescence quenching

While the dynamic behaviour in chemical conversions may generate difficulties for interpretation of single molecular effects on the fluorescence rate constants, the presence of different xanthophylls illustrates the leaf’s different strategies to cope with excessive energy. Maximum photoprotection could in this sense be covered by a combination of pigments properly bound within their respective LHC sites, which structurally remodels the LHCs and the antenna complex as a whole (Ware et al. 2016). A possible explanation is that the presence of different xanthophylls can increase the density of quenching sites for a given concentration, enhancing the likelihood of correct pigment binding at specific, as also previously suggested by Leuenberger et al. (2017). It has also been proposed that when two xanthophylls such as Zea and Lut are in close proximity to each other and to chlorophyll, they can cooperatively enhance quenching beyond what either can achieve alone (Horton et al. 2005; Johnson et al. 2009). In fact, some studies even implicate Neo in the mechanism of photoprotection, by modulating Vio binding affinity in LHCs, accelerating the Vio-to-Ant step of the VAZ cycle, and promoting transient NPQ (Wang et al. 2017). While the role of individual xanthophylls is left to be explored (perhaps through other kind of experiments), the need for Zea availability is generally shown to be essential under light stress conditions (Dall’osto et al. 2012; García-Martínez et al. 2025; Nilkens et al. 2010). This can also be deduced from this experiment. Moreover, it was shown that Zea accumulation explains best the fluorescence decrease along the transient of 180 s based on correlation, except in the case of shade grown *M. alba* leaves (Fig. S7 in *Supplementary Information*). Despite that, the F decline can be additionally due to other mechanisms, e.g. such as the different reaction in the opening of PSI and PSII RCs, the role of newly formed quencher molecules in the time frame of strong F decline, with matching half-times of the processes, should be considered. Zea has been accepted as the strongest potential quencher, as quantitative models suggest on a per-molecule basis that Zea is about 10 times more effective in dissipating energy than Lut (Leuenberger et al. 2017), and 3 times more effective than Ant (Short et al. 2023). Other recent work pointed out the quenching role of Zea using annihilation-free transient absorption measurements providing direct spectroscopic evidence that qE involves excitation energy transfer (EET) from Chl Qy to the Zea S1 state (Lee et al. 2024). While we acknowledge that the individual causal relationships between pigments and their effect on the rate constant of fluorescence is difficult to illustrate during the complex dark-to-high-light transient, the absorption behaviour at sub-second resolution (Fig. 8) may provide new paths for analysis. It is crucial to hereby appreciate that the quick behaviour in the early seconds shows the importance of heterogeneous absorbance components (Fig. 8), while the apparent absorbance increases developed after 20–30 s are dominated by one component (in all cases here: 535 nm). A conclusion that can be drawn from this is that available Xan pool does not necessarily mean that the activation of heat dissipation is triggered for all newly formed Xan pools. Slower triggering of different energy dissipation mechanisms may perhaps occur upon the gradual increase of triggers, shifting the spectral behaviour. For example, tomato plants have shown a first 520-nm component under excessive light, which can be red-shifted under drought stress (García-Martínez et al. 2025), while other species have shown a 530 nm peak being shifted to a 550 nm peak within 20 min of excessive light (Van Wittenberghe et al. 2019).

### Quick LxL cycle

A novel result in our work is that we were able to quantify very dynamic Lx pools, resulting proportionally even more dynamic than the Lut pool for example (Table 1). The fast pigment sampling protocol moreover revealed an active LxL cycle within the first seconds for sun leaves of *Q. ilex* (Fig. 6f), before VAZ cycle pigments were fully converted. Hence, Lx and Lut oscillations preceded Zea accumulation (Figs. 3 and 4), implying a temporal division of labor: Lut may mitigate initial excitonic pressure, while Zea pools are formed in later step. Such a mechanism would explain why Lut-deficient mutants exhibit NPQ defects despite normal VAZ cycle operation (Li et al. 2009). Our results further suggest that Lut may act as an early responder to stress, but may also decrease again when Ant and Zea are being formed (Fig. 10). Lut and Lx accumulation results eventually lower in the steady-state pool at 180 s (Figs. 3, 4 and 10), except in the case of shade leaves of *M. alba*, where both Lx and α-Car showed significant increases in relative pool size at steady-state (Figs. 3c and 4c). This could highlight the importance of the LxL cycle activity for shade leaves. As argued by Vialet-Chabrand et al. (2017), shade leaves can be prone to sudden sun flecks, and therefore more frequently facing contrasting low versus high light intensities. Such extreme exposure conditions may require an extremely fast mechanism to avoid sudden damage to the antenna.

Both sun and shade leaves however eventually invest in Zea accumulation when exposed to several minutes of excessive light (Fig. 3, with the exception of *M. alba* shade leaves), agreeing with many studies demonstrating the accumulation of Zea under high light (Grebe et al. 2020; Johnson et al. 2009; Nilkens et al. 2010; Ruban 2016; Verhoeven 2014), and as was discussed before. These observations collectively emphasize that prompt photoprotection may arise from pigment synergism/multiple potential energy quenchers, with each molecule contributing distinct temporal and functional roles. Again, while our pigment analyses capture the immediate interconversions, it does not address longer-term structural reconfigurations of the antenna and further activated quenching role of the different xanthophylls. Several of these processes likely unfold over slower timescales and should be examined under diurnal light profiles to fully understand how steady-state antenna modifications develop under certain stoichiometric changes.

### Dynamic α- and β-branch balancing and de novo synthesis

In addition to the common observed xanthophyll cycles, highly heterogeneous fluctuations in α-Car and β-Car were also observed during the first 20 seconds of illumination. For example, in the case of *M. alba* shade leaves the α-Car pool increased significantly, while the VAZ and β-Car pools depleted (Figs. 3c, 4c and S4 in *Supplementary Information*) In addition, total α-branch trends mirrored sometimes the β-branch trends in the early transient phase (Fig. 7), suggesting cross-talk and dynamic precursor allocation between α- and β-branch pathways. Indeed, the biosynthesis of both xanthophyll branches is not fully independent. The precursor for Lut formation is α-Car, which can accumulate at the expense of β-Car—the precursor of the VAZ cycle—in leaves and fruits of several species (Simkin et al. 2022). This reciprocal behaviour supports the idea of dynamic competition for shared precursors between branches. Sun- and shade-adapted leaves maintain distinct carotene baselines, with α-Car/Chl a generally higher in shade and β-Car/Chl a higher in sun leaves (Dörken and Lepetit 2018). However, the balance or trade-off between these α- and β-branch conversions during rapid light transitions remains poorly understood, particularly in the context of short-term antenna acclimation. These findings challenge the assumption that carotenes act merely as static precursors, instead implicating them as active participants in the immediate regulation of light stress. Genetic evidence shows that β-Car flux directly shapes photoprotective capacity, as overexpression of β-carotene hydroxylase expands the xanthophyll-cycle pool and enhances stress tolerance (Davison et al. 2002). In addition, β-Car is particularly susceptible to singlet oxygen-mediated oxidation under excess light, leading to rapid turnover and the formation of reactive apocarotenoids such as β-cyclocitral that act as stress signals (Havaux 2014). While such effects were described in longer-term acclimation contexts, our results suggest that carotene rebalancing and possibly the earliest phases of oxidative signaling may already be initiated within seconds of illumination.

Overall, sun and shade leaves appear to employ slightly different stoichiometric acclimation strategies: shade leaves exhibit stronger LxL cycle modulation (Fig. 6), while sun leaves exhibit faster VAZ cycle kinetics (Fig. 4) but maintain smaller dynamic pools (Table 1), reflecting their pre-acclimation to high light (Demmig-Adams 1998). Dark-state pools of pigments, which were shown different between the different species and light conditions (Table S3 in *Supplementary Information*), may also have contributed to the early mechanisms that are rapidly engaged upon light exposure; for example, the lower dynamic Zea pool observed in sun-grown *M. alba* leaves (Table 1) could reflect the appreciable amount of Zea already present at *t* = 0 (Fig. S3).

Together, these results highlight that early carotenoid dynamics, especially the interplay between α- and β-branches, and also the mirror trends seen for Vio-Neo (Fig. 5) and α-car, Lx and Lut (Fig. 6) constitute critical and previously underappreciated components of short-term photoprotective acclimation. These findings also suggest that the classical representation of carotenoid conversions shown in Fig. 1c may be rather conservative regarding their response to sudden excessive light exposure. While the scheme depicts well-established directional steps within the VAZ and LxL cycles, such reciprocal behaviour demonstrates that the flux between these pools may oscillate in both directions.

## Conclusions

This study demonstrates that the first steps of plants to acclimate to sudden high light exposure are underpinned by rapid, dynamic adjustments in the entire α- and β-carotenoid branches, beyond the classic VAZ and LxL cycle schemes. Sampling for pigments at high temporal resolution, we captured previously underappreciated pigment dynamics that challenge the classical view of slower xanthophyll cycle activation. For example, our findings provide evidence of the rapid de novo synthesis and conversion of lutein, respectively from α-carotene and lutein epoxide, within the first seconds of illumination. The highly heterogeneous pigment fluctuations observed in this initial phase (0–20 s) are indicative of a dynamic, cascading mechanism involving the conversion, exchange and mobilisation of carotenoids between α- and β-branches to accommodate the antenna for energy dissipation. Within species and growing conditions we could observe variable pool dynamics. The temporal resolution of the spectral acquisition and parallel pigment dynamics allowed us further to propose two distinct processes, i.e. (1) an EET phase in which multiple modelled Gaussians can occur (0–30 s), and (2) a triggered activation of regulated heat dissipation shown by the enhancement of a dominating Gaussian peak at 535 nm. The latter is suggested to demonstrate a vibrationally hot ground state from red-shifted Ant or Zea, based on the matching spectral trends and pigment kinetics in the prior EET phase. These results show that in vivo spectroscopy at high temporal resolution may offer new avenues for understanding antenna regulation, improving dynamic NPQ models and providing tools for in vivo early stress detection.

## Electronic supplementary material

Below is the link to the electronic supplementary material.

Supplementary Material 1

## Data Availability

Raw and processed data are available open access through the Zenodo repository (doi: 10.5281/zenodo.16568809).
