# Supplementary material for: Quick conversions and de novo synthesis within the entire α- and β-carotenoid branches during non-steady-state light transients
Source: Photosynth Res. 2026 Jun 6;164(3):33. doi: 10.1007/s11120-026-01221-5 (PMC13242464; doi:10.1007/s11120-026-01221-5)
Supplement: Supplementary file 1 — Supplementary Material 1 [file 11120_2026_1221_MOESM1_ESM.docx]

**Supporting Information**

**Table S1** Ternary gradient elution used for pigment separation. The solvents used were methanol (MeOH), MilliQ water (H_2_O) and methyl tert-butyl ether (MTBE). After 47 minutes of gradient elution, percentages remained stable for re-equilibration until the injection of the next sample.

| **Time (min)** | **0** | **12** | **20** | **30** | **45** | **47** |
| --- | --- | --- | --- | --- | --- | --- |
| **MTBE(%)** | 5 | 5 | 14 | 25 | 55 | 5 |
| **H2O(%)** | 5 | 0 | 0 | 0 | 0 | 5 |
| **MeOH(%)** | 90 | 95 | 86 | 75 | 45 | 90 |

**Table S2.** Chromatographic and spectroscopic characteristics of carotenoids and chlorophylls quantified in leaf samples. Peaks are numbered by elution order (see chromatogram in Figure S1).

a*, identified using authentic standards; Neoxanthin was identified by purification from spinach leaf and lutein-5,6-epoxide from sweet bay laurel leaf; b Peak ratio is % III/II for carotenoids (Britton, 1995); s, shoulder

| **Peak** | **Retention time** | **Pigment^a^** | **UV-Vis absorption maxima (nm)** |  | **% III/II^b^** |
| --- | --- | --- | --- | --- | --- |
| **1** | 11.9 | *Violaxanthin | 415,438,468 |  | 95 |
| **2** | 12.8 | Neoxanthin | 414,435,463 |  | 80 |
| **3** | 14.4 | Lutein-5,6-epoxide | 415,437,468 |  | 85 |
| **4** | 16.9 | *Antheraxanthin | 420(s),443,470 |  | 55 |
| **5** | 18.4 | *Chlorophyll b | 468,650 |  | - |
| **6** | 19.8 | *Lutein | 418,443,471 |  | 65 |
| **7** | 22.4 | *Zeaxanthin | 430,449,476 |  | 35 |
| **8** | 23.6 | *Chlorophyll a | 421,665 |  | - |
| **9** | 35.1 | *α-carotene | 420,445,473 |  | 55 |
| **10** | 38.5 | *β-carotene | 426(s),451,473 |  | 25 |

**Table S3.** Mean values of dark-adapted pigment pools (± standard error, *n*=3) for the carotenes and xanthophylls, normalized over the Chl a pool (for inter-leaf comparison). Units are shown in mmol mol^-1^ to facilitate interpretation.

|  | ***C. australis*** | | ***M. alba*** | | ***Q. ilex*** | |
| --- | --- | --- | --- | --- | --- | --- |
| **Pool size in darkness (mmol mol^-1^)** | **shade** | **sun** | **shade** | **sun** | **shade** | **sun** |
| **[Neo/Chl.a]** | 40.0 ± 0.3 | 30 ± 1 | 50 ± 2 | 60 ± 1 | 40 ± 1 | 50 ± 20 |
| **[Vio/Chl.a]** | 20 ± 1 | 30 ± 7 | 120 ± 2 | 120 ± 10 | 20 ± 5 | 50.0 ± 0.7 |
| **[Ant/Chl.a]** | 20 ± 2 | 20 ± 9 | 20 ± 2 | 6 ± 0.2 | 10 ± 7 | 3.0 ± 0.2 |
| **[Zea/Chl.a]** | 1.0 ± 0.2 | 0.9 ± 0.9 | 20 ± 3 | 20 ± 1 | nd | 7.0 ± 0.1 |
| **[β-car/Chl.a]** | 100 ± 4 | 110 ± 10 | 110 ± 10 | 140 ± 2 | 70 ± 4 | 120 ± 3 |
| **[α-car/Chl.a]** | 20 ± 0.8 | 6 ± 1 | 4.00 ± 0.03 | 2.00 ± 0.04 | 60.0 ± 0.6 | 10 ± 1 |
| **[Lut/Chl.a]** | 190 ± 4 | 200 ± 3 | 220 ± 8 | 180 ± 4 | 160 ± 4 | 150 ± 2 |
| **[Lx/Chl.a]** | 0.6 ± 0.3 | nd | 3.0 ± 0.4 | 4.0 ± 0.5 | 10 ± 3 | 10 ± 1 |


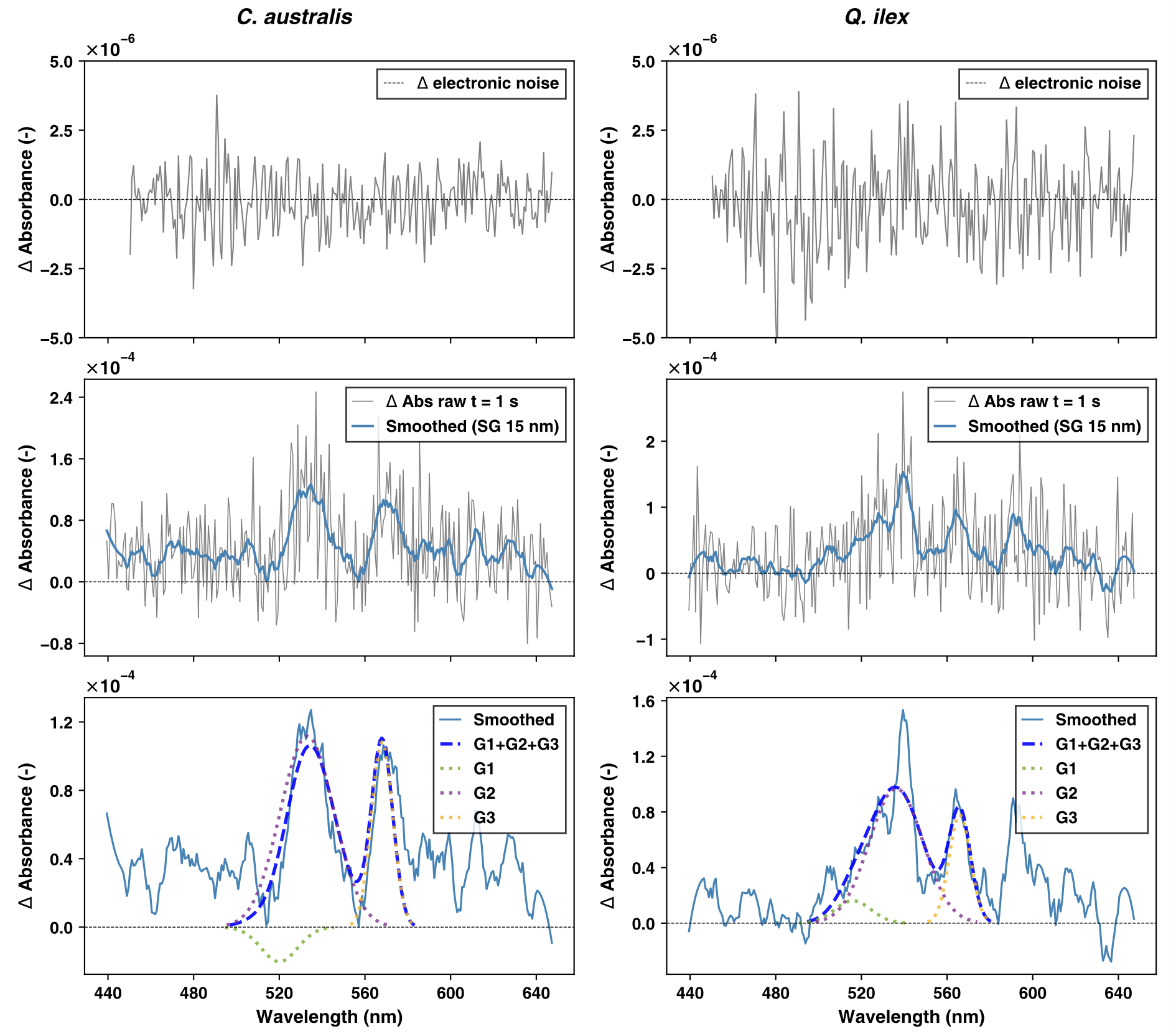


**Figure S1.** Difference signals in dark (electronic noise, panel a) and at the acquisition time of 1 s exposure of 1000 µmol m^-2^ s^-1^ (panel b) for *C.australis* and *Q. ilex*. The raw absorbance change spectrum (in grey) is shown together with the corresponding smoothed spectrum (in blue) for illustrative purposes (panel b) indicating the underlying signal which is fitted by the three constrained Gaussian components G1, G2 and G3, with means around 520, 535 and 560 nm (panel c). The fitting analysis was performed on the raw spectral data.

**
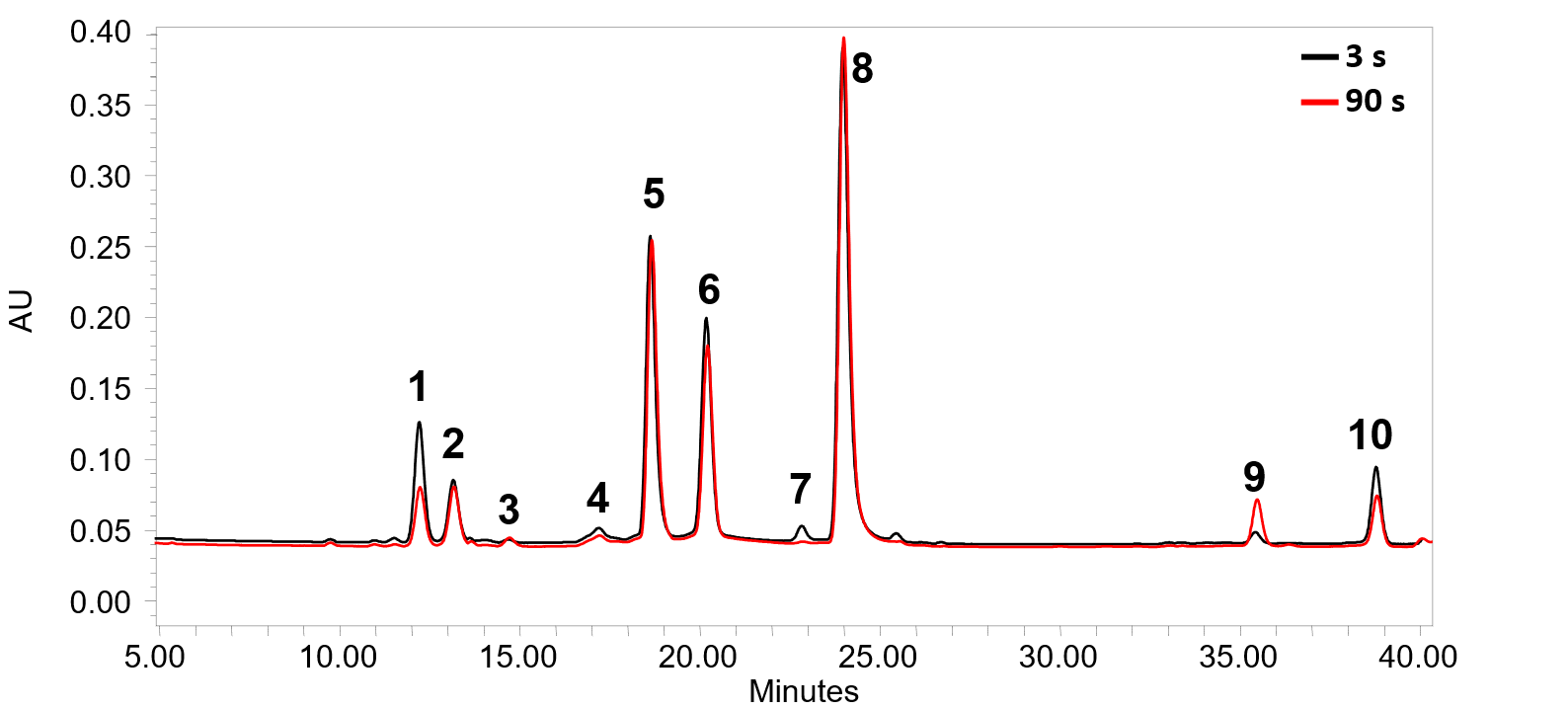
**

**Figure S2.** Representative chromatogram of a pigment extract corresponding to *M. alba* shade leaves at two different time points (3 s, black; 90 s, red). The profile is a MaxPlot chromatogram (each pigment shown at its individual λ maxima). Peak identification is described in Table S2. AU, Absorption units.


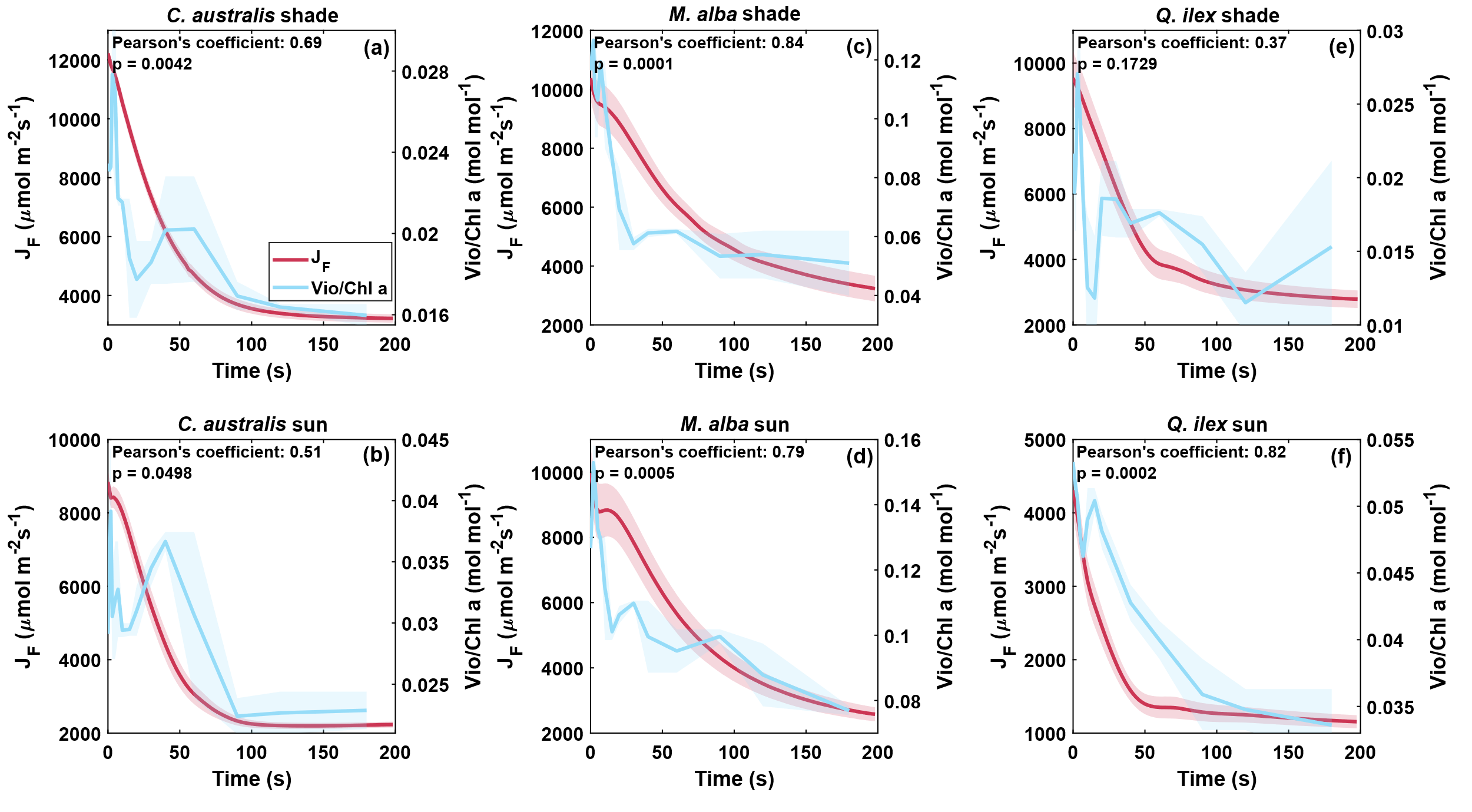


**Figure S3.** Dynamics of fluorescence quenching (J_F_, *n*=6) and violaxanthin pool (Vio/Chl a, *n*=3) under high light exposure in sun- and shade-adapted leaves of *C. australis*, *M. alba*, and *Q. ilex*. Red lines represent J_F_ (left y-axis), while blue lines represent the molar ratio of violaxanthin to chlorophyll a (Vio/Chl a, right y-axis) over time during high light treatment. Shaded areas indicate ± standard error. Pearson’s correlation coefficients between J_F_ and Vio/Chl a are reported in each panel along with their associated p-values.

In sun-grown leaves, moderate to strong correlations were observed: *C. australis* (R=0.5145, p=0.0498), *M. alba* (R=0.7860, p=0.0005), and *Q. ilex* (R=0.8192, p=0.0002). Similarly, significant correlations were detected in shade-grown leaves of *C. australis* (R=0.6927, p=0.0042) and *M. alba* (R=0.8393, p=0.0001). In contrast, *Q. ilex* shade-grown leaves displayed a weaker and statistically non-significant correlation (R=0.3714, p=0.1729), suggesting a reduced reliance on VAZ cycle-mediated quenching in this context.


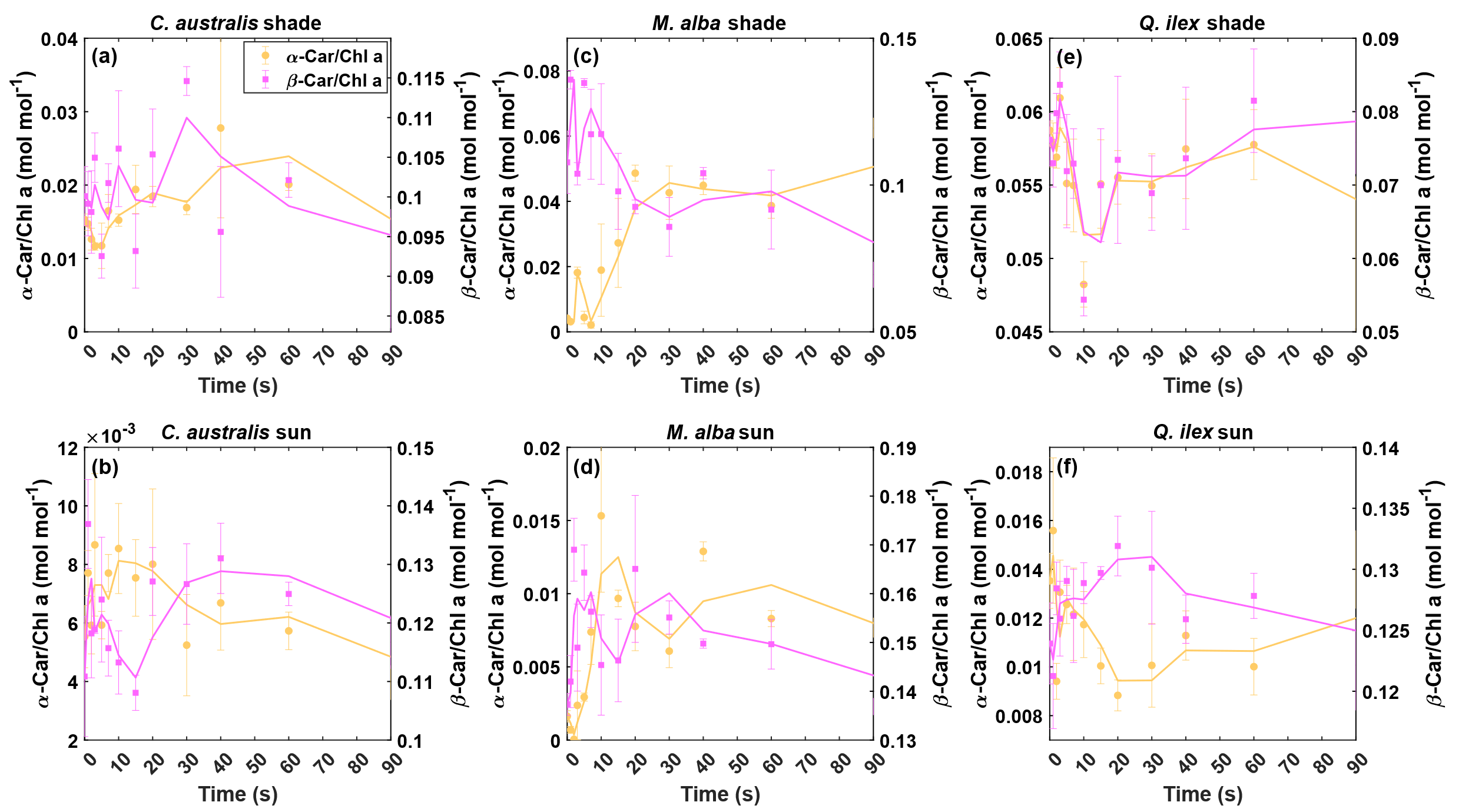


**Figure S4.** Transient trends of α-carotene (α-Car, yellow, left y-axis) and β-carotene (β-Car, pink, right y-axis) in the first 90 s of high light illumination in shade (panels a,c,e) and sun-adapted (panels b,d,f) leaves of *C. australis, M. alba* and *Q. ilex*. Data shown are the mean pigment concentration (normalized over Chl a, mol mol^-1^, *n=3*) ± standard error, and the trend line shows the 2-period moving mean. Note that in this figure the axis do not represent the same range of variation.

**
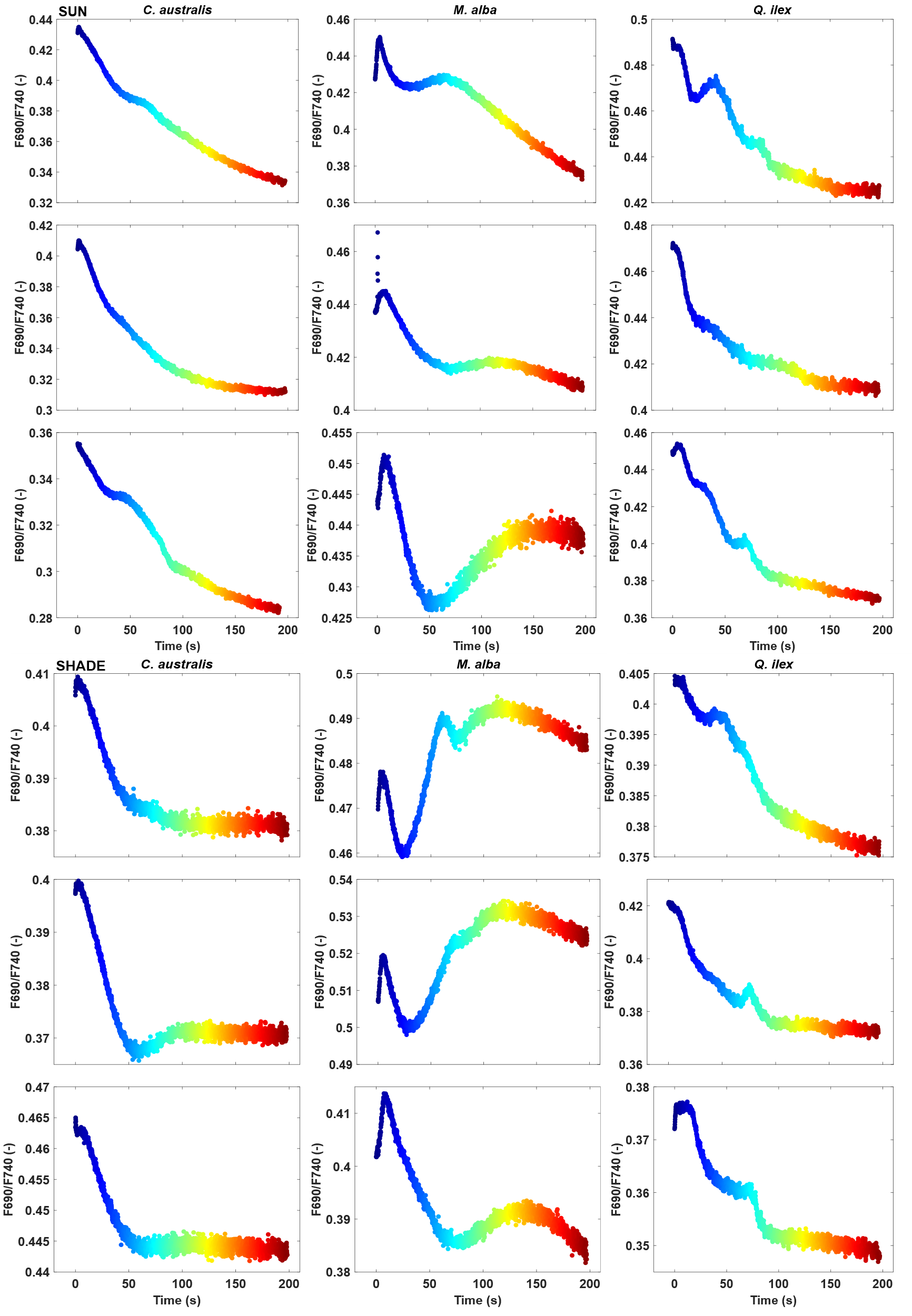
**

**Figure S5.** Time-resolved changes in the red (F690) and far-red (F740) fluorescence ratio peaks for three different sun- and shade-grown leaves from *C. australis*, *M. alba* and *Q. ilex* during high light exposure.


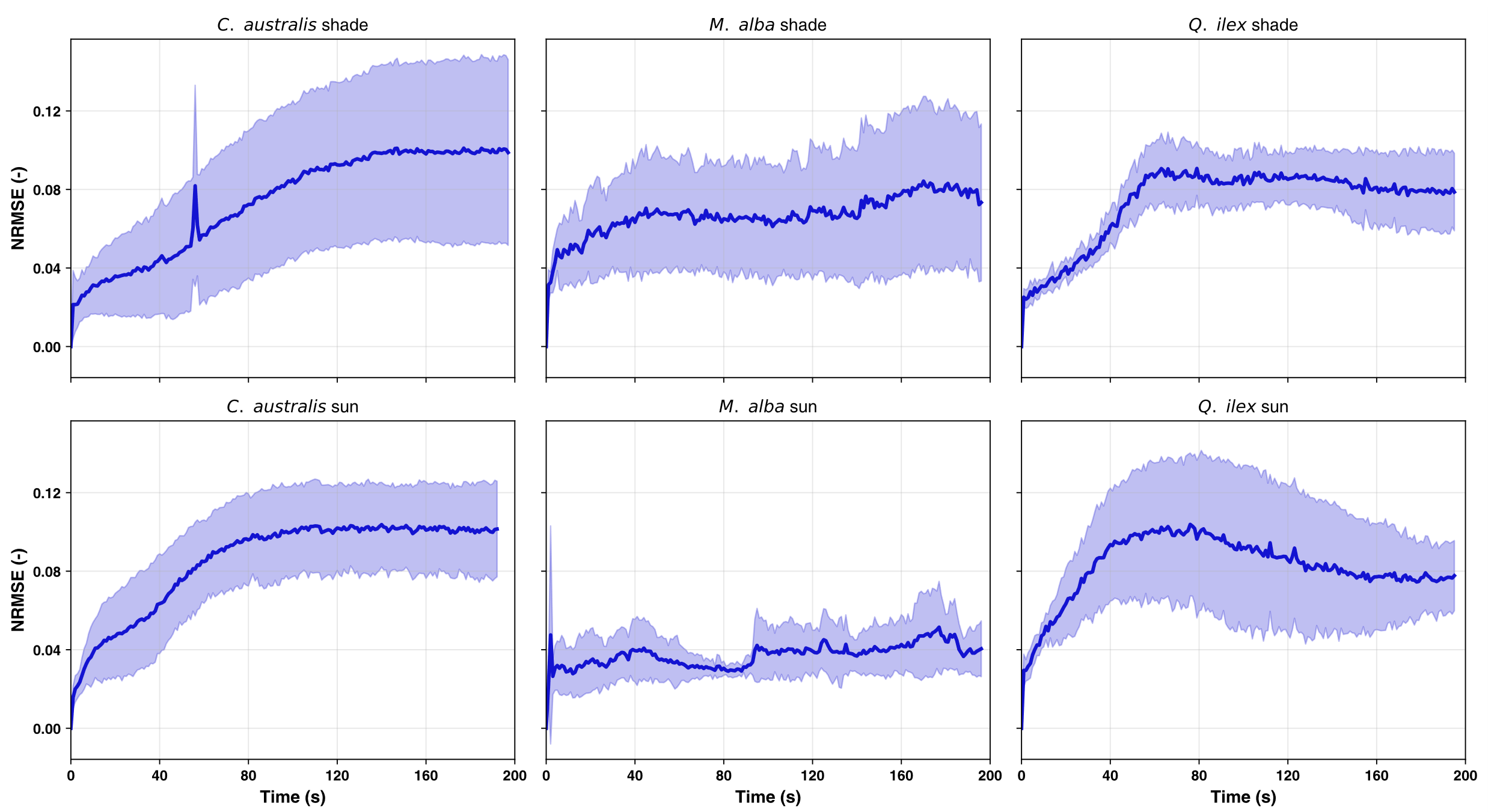


**Figure S6.** Normalized root mean square error (NMRSE, mean ± standard error, *n=6*) of the Gaussian peak fitting for the transients under high light exposure in sun- and shade-adapted leaves of *C. australis, M. alba*, and *Q. ilex*.


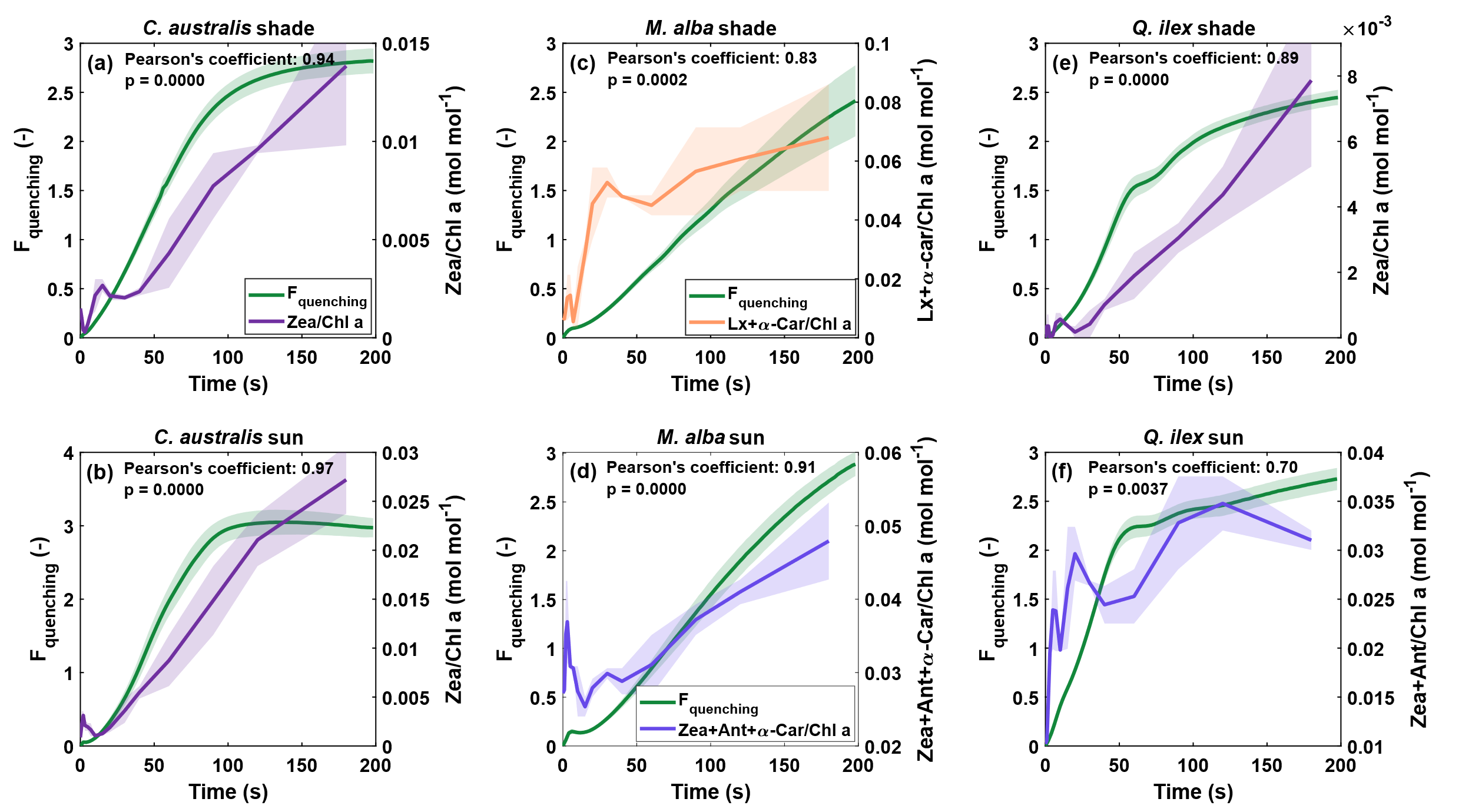


**Figure S7.** Correlation between fluorescence quenching calculated from equation 2 (F_quenching_) (*n*=6) and xanthophyll-cycle pigment (normalized over Chl a, *n*=3) accumulation during high light exposure in sun- and shade-acclimated leaves of *C. australis*, *M. alba* and *Q. ilex*. For each case, the sum of all carotenoids that were significantly at t=180 s (see statistics in Fig. 3) was used in the analysis. Green lines represent F_quenching_ (left y-axis), while colored lines show the sum of the specific carotenoids (normalized to Chl a) significantly increased at t=180 s in each case (right y-axis). Shaded areas indicate ± standard error. Pearson's correlation coefficients between F_quenching_ and carotenoid metrics are shown for each condition with associated p-values.


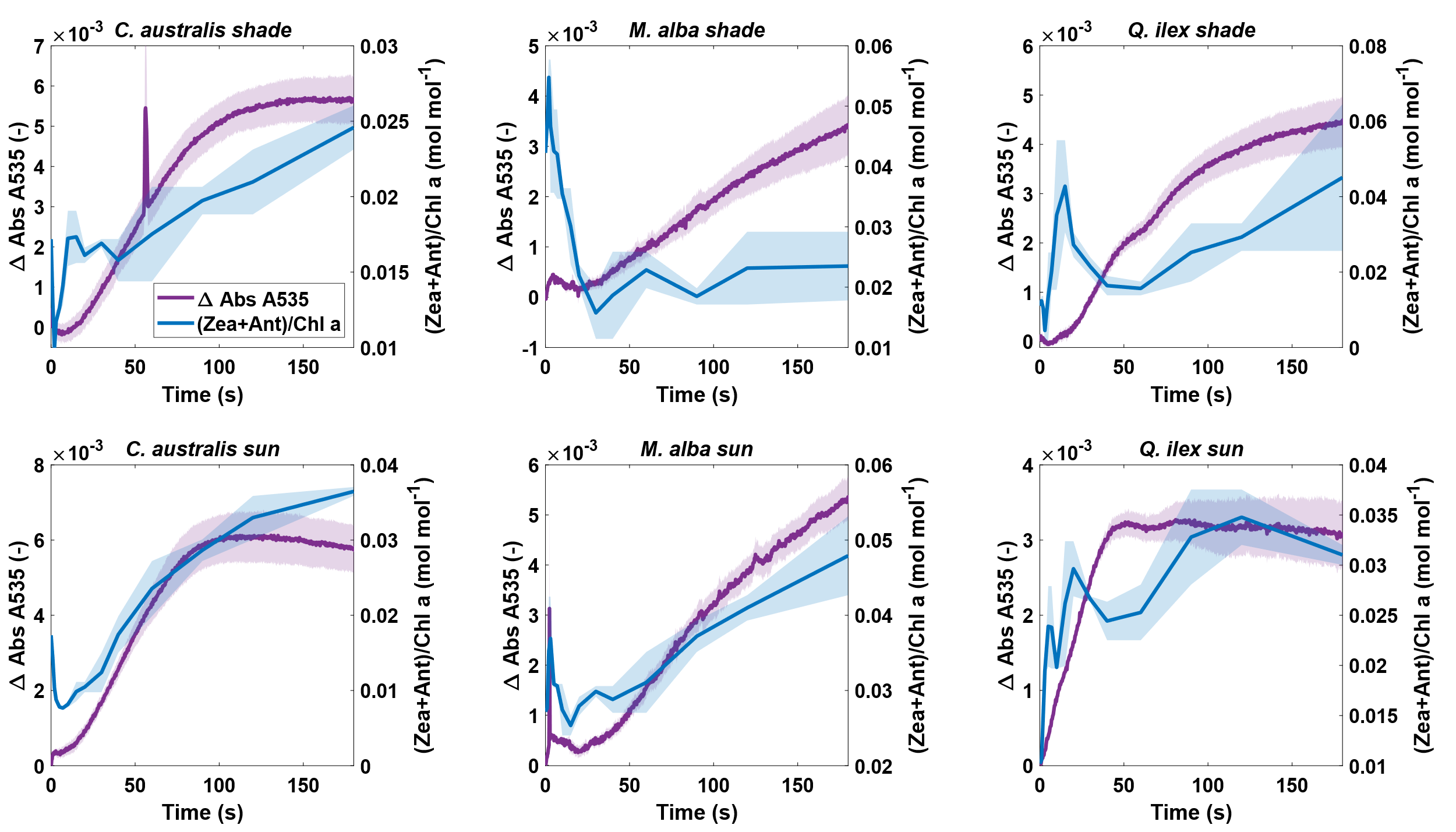


**Figure S8.** Correlation between ΔAbs peak at 535 nm (-) (*n*=6) and the sum of zeaxanthin (Zea) and antheraxanthin (Ant) pigments (normalized over Chl a, *n*=3) accumulation during high light exposure in sun- and shade-acclimated leaves of *C. australis*, *M. alba* and *Q. ilex*. Purple lines represent ΔAbs 535 (left y-axis), while blue lines show the sum of Zea and Ant (normalized to Chl a) (right y-axis). Shaded areas indicate ± standard error.
